# Programmable and Reversible Integrin‐Mediated Cell Adhesion Reveals Hysteresis in Actin Kinetics that Alters Subsequent Mechanotransduction

**DOI:** 10.1002/advs.202302421

**Published:** 2023-10-17

**Authors:** Zheng Zhang, Hongyuan Zhu, Guoqing Zhao, Yunyi Miao, Lingzhu Zhao, Jinteng Feng, Huan Zhang, Run Miao, Lin Sun, Bin Gao, Wencheng Zhang, Zheng Wang, Jianfang Zhang, Ying Zhang, Hui Guo, Feng Xu, Tian Jian Lu, Guy M. Genin, Min Lin

**Affiliations:** ^1^ The Key Laboratory of Biomedical Information Engineering of Ministry of Education School of Life Science and Technology Xi'an Jiaotong University Xi'an 710049 P. R. China; ^2^ Bioinspired Engineering and Biomechanics Center (BEBC) Xi'an Jiaotong University Xi'an 710049 P. R. China; ^3^ Department of Medical Oncology First Affiliated Hospital of Xi'an Jiaotong University Xi'an 710061 P. R. China; ^4^ Department of Endocrinology Second Affiliated Hospital of Air Force Military Medical University Xi'an 710038 P. R. China; ^5^ Department of Hepatobiliary Surgery First Affiliated Hospital of Xi'an Jiaotong University Xi'an 710061 P. R. China; ^6^ Department of Gynaecology and Obstetrics of Xijing Hospital， Fourth Military Medical University 710054 Xi'an P. R. China; ^7^ Xijing 986 Hospital Department Fourth Military Medical University Xi'an 710054 P. R. China; ^8^ State Key Laboratory of Mechanics and Control of Mechanical Structures Nanjing University of Aeronautics and Astronautics Nanjing 210016 P. R. China; ^9^ Department of Mechanical Engineering & Materials Science Washington University in St. Louis St. Louis MO 63130 USA; ^10^ NSF Science and Technology Center for Engineering Mechanobiology Washington University in St. Louis St. Louis MO 63130 USA

**Keywords:** actin alignment, cell adhesion, mechanical memory, mechanical microenvironment, mechanotransduction

## Abstract

Dynamically evolving adhesions between cells and extracellular matrix (ECM) transmit time‐varying signals that control cytoskeletal dynamics and cell fate. Dynamic cell adhesion and ECM stiffness regulate cellular mechanosensing cooperatively, but it has not previously been possible to characterize their individual effects because of challenges with controlling these factors independently. Therefore, a DNA‐driven molecular system is developed wherein the integrin‐binding ligand RGD can be reversibly presented and removed to achieve cyclic cell attachment/detachment on substrates of defined stiffness. Using this culture system, it is discovered that cyclic adhesion accelerates F‐actin kinetics and nuclear mechanosensing in human mesenchymal stem cells (hMSCs), with the result that hysteresis can completely change how hMSCs transduce ECM stiffness. Results are dramatically different from well‐known results for mechanotransduction on static substrates, but are consistent with a mathematical model of F‐actin fragments retaining structure following loss of integrin ligation and participating in subsequent repolymerization. These findings suggest that cyclic integrin‐mediated adhesion alters the mechanosensing of ECM stiffness by hMSCs through transient, hysteretic memory that is stored in F‐actin.

## Graphical abstract

The shorter cycle period of integrin‐mediated adhesion can activate the hMSCs' mechanosensing by inducing F‐actin alignment, which then results in nuclear flattening, finally promoting YAP nuclear translocation, and these effects accumulate over repeated cycles. Conversely, the longer cycle period of integrin‐mediated adhesion has no significant effect on mechanosensing.

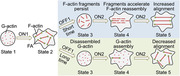

## 1 Introduction

Integrin‐mediated adhesions that link cells and extracellular matrix (ECM) serve as bidirectional hubs that transmit the mechanical signals^[^ ^1^ ^]^ and thus drive cell migration,^[^ ^2^ ^]^ proliferation,^[^ ^3^ ^]^ differentiation,^[^ ^4^ ^]^ and metastasis.^[^ ^5^ ^]^ Much of what is known about how mechanical signals affect cells arises from experiments in which a cell‐adhesive ligand such as RGD peptide is presented atop a hydrogel substrate. Although such systems have provided valuable insight into how mechanosensing is driven by the spacing,^[^ ^6^ ^]^ strength,^[^ ^7^ ^]^ tether length,^[^ ^8^ ^]^ and order/disorder^[^ ^9^ ^]^ of adhesion ligands, they have not captured the often cyclic dynamics of adhesions observed over development, regeneration, and disease progression. Such cyclic ligation affects subsequent kinetics,^[^ ^10^ ^]^ but the cytoskeletal basis of these effects and their influence on cell behavior remains unknown.

For example, mechanical loading can alter actin depolymerization kinetics^[^ ^11^ ^]^ and network structures,^[^ ^12^ ^]^ two factors critical to the ways that cells transduce force and stiffness.^[^ ^13^ ^]^ Changes in a cell's mechanical environment can fluidize or fracture the cytoskeleton, and lead to the formation of actomyosin clusters that are associated with changes to actin kinetics.^[^ ^14^ ^]^ We therefore hypothesized that cyclic adhesion would affect actin kinetics and hence mechanosensing of human mesenchymal stem cells (hMSCs) in a way that depended upon the mechanical history of the adhesions. With the problem in mind, a cell culture system with user‐defined cyclic regulation of cell adhesion is essential for a better understanding of the biological processes in vivo.

Despite this interest, there are only a few examples of approaches in which cell adhesion can be reversibly and cyclically regulated, none without their limitations. For example, Wong et al. reported a magnetically actuated dynamic cell culture platform, where RGD‐coated magnetic nanoparticles (MNPs) could be presented or concealed by controlling the upward/downward magnetic attraction to inhibit/enhance cell adhesion.^[^ ^15^ ^]^ Nevertheless, this approach is only applicable to very soft hydrogels, since the stiff hydrogel matrix is less deformable by the magnetic‐driven MNPs. Another recent study showed that reversibly switching a polypyrrole array between hydrophobicity and hydrophilicity via an electrochemical method can cyclically regulate cell adhesion.^[^ ^16^ ^]^ However, electrical stimulation may not be biocompatible for cells. Intriguingly, DNA can be a good candidate for anchoring bioadhesive ligands to the substrates and regulating their interaction with cells due to its highly programmable nature.^[^ ^17^ ^]^ For instance, a recent work reported a DNA‐mediated molecular system that can be programmed to control the dynamics, spatial positioning, and combinatorial synergies of biological signals to direct cell behaviors.^[^ ^18^ ^]^

Here, we developed a DNA‐driven platform that enabled cyclic integrin‐mediated adhesion and queried how cyclic adhesion regulates the mechanosensing of human mesenchymal stem cells (hMSCs). In this approach, the cell‐adhesive peptide RGD could be dynamically presented or removed via the DNA hybridization and the toehold‐mediated strand displacement reaction to provide cyclic attachment and detachment stimuli for hMSCs. Our observations show that a shorter cycle period (*T* = 1 or 2 h) can activate the hMSCs mechanosensing by inducing F‐actin alignment, which then results in nuclear flattening, finally promoting YAP nuclear translocation, and these effects accumulated over repeated cycles. Conversely, the longer cycle period (*T* = 6 h) had no significant effect on mechanosensing. To elucidate the reasons for the distinct response of hMSCs mechanosensing to cyclic integrin‐mediated adhesion, we established a molecular clutch‐based actin dynamic model by assuming that accumulative F‐actin dynamics contribute to the distinct cellular mechanotransduction. We tested the model by examining its predictions in reproducing the experimental results. We further verify the generality of the established model by predicting the role of matrix stiffness, myosin contractility, and F‐actin degradation rate on cellular responses to cyclic integrin‐mediated adhesion. These findings highlight that cyclic attachment‐detachment stimuli can alter stem cell perception of exogenous biophysical inputs, such as ECM stiffness, thereby independently regulating the mechanosensing of hMSCs.

## 2 Results and Discussion

### 2.1 DNA Hybridization and Toehold‐Mediated Strand Displacement Enables Programmable, Reversible Ligation of Cells

**Figure 1:**
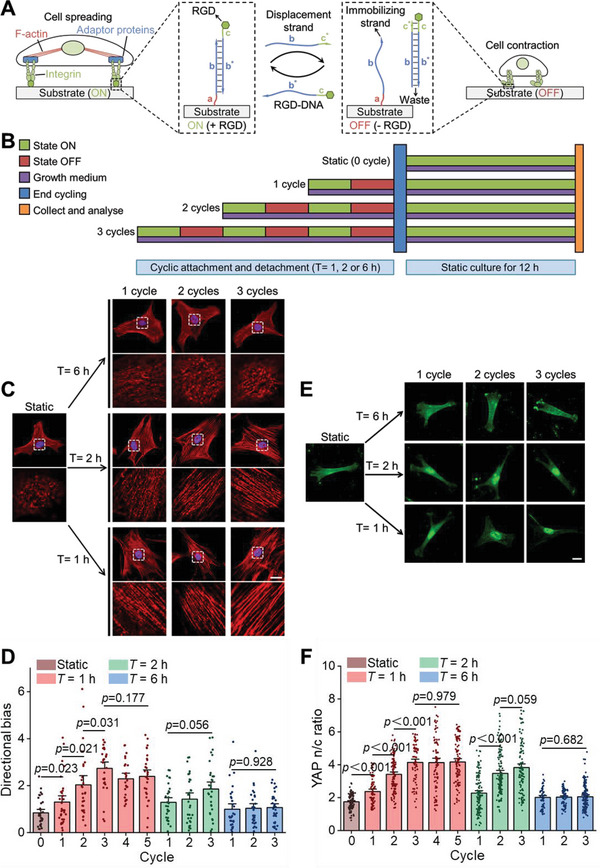
Cyclic integrin‐mediated adhesion modulates hMSCs mechanosensing. A) Schematic of cyclic integrin‐mediated adhesion enabled by a DNA‐driven molecular system. The PEG hydrogel substrate can be switched from the “ON” state (presenting RGD peptide) to the “OFF” state (without RGD peptide) through the addition of the displacement strand and can be switched back to “ON” through the addition of the RGD‐DNA molecules. B) Dosing of cyclic integrin‐mediated adhesion applied to hMSCs cultured on hydrogels in growth media (purple). hMSCs experienced prescribed numbers (1–5) and periods (*T* = 1, 2, or 6 h) of “ON” (green)/“OFF” (red) cycles, and were cultured subsequently in the “ON” state for 12 h in growth media prior to collection and analysis (yellow). Control samples were cultured in the “ON” state for 12 h in growth media without cyclic attachment‐detachment (Static). C) Top: Representative immunofluorescence images of F‐actin (red) and nuclei (blue) following prescribed doses of cycles. Scale bar, 20 µm. Bottom: Enlargements of F‐actin organization in the apical region of the nucleus (white dashed regions denoted in the top row). D) Quantification of actin directional bias (from left to right *n* = 34, 32, 29, 29, 25, 28, 31, 31, 31, 31, 31, and 32 cells; *p* values were obtained using one‐way ANOVA followed by Tukey's post hoc test, error bars are mean ± s.e.m). E) Representative images of YAP immunofluorescence (green) in response to prescribed doses of cycles. Scale bar, 20 µm. F) Quantification of YAP nuclear‐to‐cytoplasmic ratios as a function of doses of cycles (from left to right *n* = 112, 69, 116, 75, 81, 97, 116, 122, 112, 59, 79, and 149 cells; *p* values were obtained using one‐way ANOVA followed by Tukey's post hoc test, error bars are mean ± s.e.m).

RGD peptide (an integrin‐binding ligand) was cyclically presented on or removed from a hydrogel substrate via DNA hybridization and toehold‐mediated strand displacement reactions to switch the substrate between adhesive and poorly adhesive states (**Figure** **1**A; Figure S1, Supporting Information; Supporting Method 1). We define the “ON” state as one where the RGD epitope is functional, resulting in cell spreading, and the “OFF” state where the signal is removed, which should inhibit spreading (Figures S2 and S3, Supporting Information). Hydrogel stiffness could be tuned independently from 3 to 30 kPa by varying concentrations of PEG‐SH with a constant concentration of PEG‐MAL (Figure S4, Supporting Information). With the increase in PEG‐SH concentration, the stiffness of hydrogel showed an increasing trend. As gel stiffness is proportional to the number of effective crosslinks,^[^ ^19^ ^]^ it indicates that the crosslinking degree of hydrogels also increased gradually. Modification with RGD did not affect the stiffness of PEG hydrogels (Figure S4, Supporting Information). To determine the effect of RGD concentration on hMSCs spreading, substrates with a wide range of RGD concentration (from 125 to 1000 µm) were fabricated (Figure S5, Supporting Information). The results demonstrated that a concentration of 500 µm resulted in the equilibrium of cell spreading. Therefore, this condition was adopted for subsequent experiments. One key advantage of this approach is that switching to the “OFF” state regenerates the free immobilizing strand, thus allowing the reintroduction of RGD‐DNA to restore the ON state (Figure S6, Supporting Information). State switching between the “OFF” and “ON” required ≈10 min (Figure S7, Supporting Information). Notably, While regenerating the surface strand for binding a second P‐DNA molecule. Thus, the substrate could be reversibly switched between “ON” and “OFF” states to provide cyclic attachment and detachment stimuli, resulting in repeated cell spreading and contraction (Figures S8–S10 and Movie S1, Supporting Information).

Regarding RGD removal, the amount of RGD released from the substrates with cells was lower than that from the substrates without cells (Figure S11, Supporting Information). This may be explained by the fact that even if the RGD‐bioactive strand hybridizes with the displacement strand and is removed from the system of presentation, such molecules with the RGD remain attached to the cells by the integrins. Given that integrins are constantly endocytosed from the cell surface.^[^ ^1a^ ^]^ Endocytosed integrins are trafficked for degradation in lysosomes or are recycled back to the plasma membrane to facilitate the generation of new adhesion sites.^[^ ^20^ ^]^ Therefore, even if the released RGD continues to attach to the integrin, it has no effect when activating the system again with fresh RGD‐DNA molecules due to endocytosis. This notion was further supported by the fact that the number and area of cells on the “ON” substrates did not change with the increase in the number of cycles (*T* = 6 h) (Figure S9, Supporting Information). To assess whether ECM secretion by cells can limit the diffusion of DNA molecules in the hydrogel, we further explored the effect of the number of cyclic adhesions on ECM secretion by cells. The substrates with no cells (control) indicate no collagen I and fibronectin present (Figure S12, Supporting Information). Notably, the cells secreted almost no collagen I and fibronectin as the number of cycles (*T* = 6 h) increased. This may be explained by negligible ECM secreted by cells over a short period of time. Next, we further determined whether the presence of cells affected RGD conjugation on the substrate. As expected, diffusion of small molecules was not restrained as the number of cyclic adhesions increased with the presence of cells on the substrate (Figure S13, Supporting Information). Fluorescent peptide tagging and fluorescence imaging were also performed to visualize the distribution of peptides on the substrates with or without cells. Results from this assay showed that RGD conjugation to the substrate is not affected by the presence or absence of cells (Figure S14, Supporting Information).

### 2.2 Cyclic Integrin‐Mediated Adhesions Promote F‐Actin Alignment and YAP Nuclear Localization

We quantified how the cyclic integrin‐mediated adhesions affected hMSCs mechanosensing by applying prescribed numbers and periods of cyclic adhesions (Figure 1B). It has been reported that 5 kPa represents a threshold for the engagement of the molecular clutch.^[^ ^21^ ^]^ Above the stiffness threshold, force loading becomes fast enough to allow talin unfolding before integrin unbinding. This leads to vinculin binding and integrin recruitment, increasing integrin binding and force transmission. To explore whether stimulation of cyclic adhesion could reinforce clutch engagement on very soft substrates (i.e., 5 kPa), we selected 5 kPa stiffness to perform the following experiments. In line with previous works,^[^ ^22^ ^]^ our experiments have shown that the YAP nuclear‐to‐cytoplasmic (n/c) ratios of the cells reached equilibrium after a 12 h culture on the continuous “ON” substrate (Figure S15, Supporting Information). To exclude the effect of mechanosensation times, we conducted a 12 h culture on the continuous “ON” substrate following all cyclic stimulation experiments. We also selected the 12 h culture on the continuous “ON” substrate as the static control group for our subsequent experiments. As expected, minimal mechanotransduction was observed in hMSCs cultured on a substrate of 5 kPa that remained in the continuous “ON” state. A relatively small actin cap formed over the cell nucleus with little directional bias (Figure 1C,D). The nuclei of these cells contained just under twice the amount of YAP (Yes‐associate‐protein, a critical outside‐in mechanotransduction mediator, can translate physical information into protein expression by localizing to the nucleus.^[^ ^23^ ^]^) as did the cytoplasm (Figure 1E,F). And nuclear localization of RUNX2 (Runt‐related transcription factor 2, a transcriptional partner of YAP, can be coactivated along with YAP to initiate osteogenesis^[^ ^24^ ^]^) was minimal (Figure S16, Supporting Information). This was true as well for cells that were regulated between cyclic attachment and detachment when the cyclic period (*T*) for 6 h.

Surprisingly, when *T* was shortened to either 2 or 1 h, the actin cap became substantially more aligned (Figure 1C,D), and the increases were observed accompanied with increasing YAP nucleus‐to‐cytoplasm (n/c) ratio (Figure 1E,F) and with increasing nuclear localization of RUNX2 (Figure S16, Supporting Information). We additionally observed that the shorter cycle period also significantly promoted hMSCs spreading (Figure S17, Supporting Information). To further confirm this finding, we verified it with two other types of integrin‐binding peptides (GFOGER and IKVAV) and obtained similar results (Figures S18 and S19, Supporting Information). Notably, the directional bias of actin cap and YAP n/c ratios reached a maximum for up to three cycles of cyclic integrin‐mediated adhesion (*T* = 1 h) (Figure 1D; Figure S20, Supporting Information). Moreover, we observed that for a certain number of cycles, shorter *T* had a more profound effect on activating hMSCs mechanosensing, and a higher number of cycles made this effect more obvious. Cyclic integrin‐mediated adhesion thereby induced distinct alterations in cellular mechanotransduction of substrate stiffness, with cyclic adhesions giving rise to YAP and RUNX2 signaling indicating osteogenic commitment that would not otherwise occur in hMSCs cultured on substrates of this stiffness. Taken together, these findings show that cyclic integrin‐mediated adhesion amplified substrate stiffness‐driven YAP signaling in hMSCs.

### 2.3 A Clutch‐Based Actin Dynamics Model Reproduces the Experimental Results

**Figure 2:**
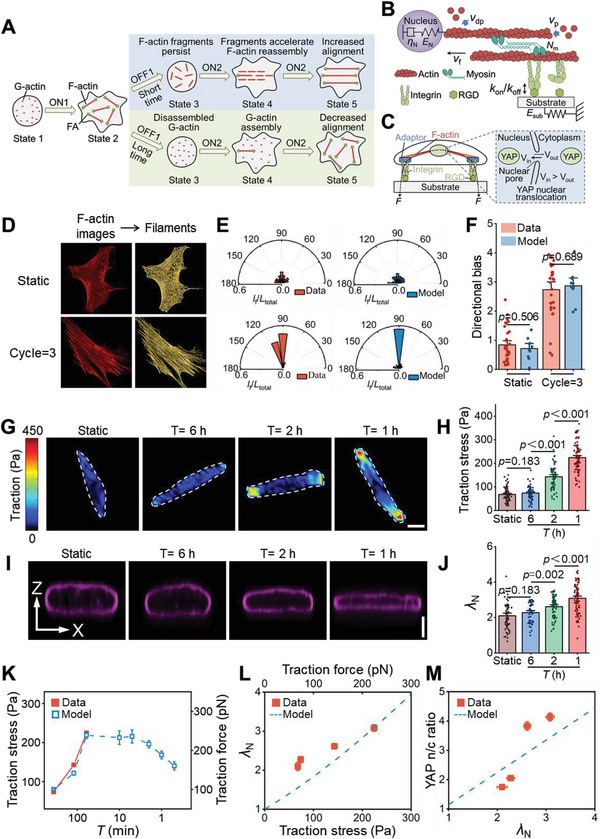
A clutch‐based actin dynamics model explains cellular mechanotransduction in response to cyclic integrin‐mediated adhesion. A) In the model, G‐actin polymerizes to form F‐actin in adherent cells (ON1). F‐actin degrades gradually during cell detachment (OFF1). The growth of F‐actin in the subsequent reattachment (ON2) can be increased by the action of residual F‐actin fragments that persist from previous detachment events. This effect decays with increasing detachment (OFF) time. B) The motor‐clutch model for a single actomyosin module. One side of the F‐actin connects to the nucleus, while the other connects to the substrate through integrin‐RGD clutches that engage and disengage with rates ***k***_**on**_ and ***k***_**off**_, respectively. Myosin drives the contraction of the F‐actin with velocity ***v***_**f**_. C) The force‐dependent YAP redistribution model. Actomyosin contractility deforms the nucleus to open nuclear pores and increases the nuclear YAP import rate. D) Representative images of F‐actin (red) in cells with static or three cycles of adhesion (*T* = 1 h), and filament traces processed using Filament Sensor software^[^ ^30^ ^]^ (yellow). E) Orientation distributions of F‐actin quantified from filament traces (left) and corresponding model predictions (right). The cumulative length of filaments in a specific direction (***L***_**i**_) was normalized to the total length of all filaments (***L***_**total**_). F) Comparison of experimental data (red) and model predictions (blue) of orientation in the experimental conditions of (E) (from left to right: *n* = 34 and 29 cells for experimental data, *n* = 10 simulations for modeling results, *p* values were obtained using one‐way ANOVA followed by Tukey's post hoc test, error bars are mean ± s.e.m). G) Representative cellular traction stress in hMSCs after static or three cycles of adhesion (*T* = 1 h) measured using traction force microscopy. The cell outlines are indicated by white dashed lines. Scale bar, 20 µm. H) Average traction stresses as a function of cyclic adhesion dosing (from left to right: *n* = 73, 69, 76, and 85 cells, *p* values were obtained using one‐way ANOVA followed by Tukey's post hoc test, mean ± s.e.m). I) Representative longitudinal sections of lamin immunostained nuclei obtained from cells with static or three cycles of adhesion (*T* = 1 h). Scale bar, 5 µm. J) Quantification of nuclear flattening as a function cyclic adhesion dosing (from left to right: *n* = 59, 51, 71, and 87 cells, *p* values were obtained using one‐way ANOVA followed by Tukey's post hoc test, error bars are mean ± s.e.m). K) Comparison of predicted (blue) and experimentally measured (red) cellular tractions with three cycles of adhesion for various cyclic periods. L) Comparison of predicted (blue dashed line) and experimentally measured (red solid dots) relationships between nuclear flattening (***λ***_**N**_) and cellular traction. M) Comparison of predicted (blue‐dashed line) and experimental (red solid dots) relationships between YAP n/c ratio and ***λ***_**N**_.

In each cycle of cell adhesion, fragments of stress fibers from the previous “ON” state persisted in the form of F‐actin after the substrate was switched to “OFF” state. We hypothesized that our observations could be explained by the participation of these fragments in repolymerization during the subsequent “ON” state in a way that speeds repolymerization kinetics (**Figure** **2**A). We adapted the motor‐clutch model of Chan‐Odde et al.^[^ ^25^ ^]^ and the cell migration simulator of Bengasser et al.^[^ ^26^ ^]^ (See modeling details in Supporting Method 2) to test this hypothesis. We modeled F‐actin fragments as motor‐clutch units^[^ ^26^ ^]^ that disassembled over time and that had lengths that were force‐dependent (Figure 2B),^[^ ^27^ ^]^ and we modeled aligned perinuclear F‐actin bundles in the actin cap as facilitating nuclear YAP accumulation (Figure 2C)^[^ ^28^ ^]^ by deforming the nucleus and thereby opening nuclear pore channels.^[^ ^29^ ^]^ To confirm experimentally this assumption, we examined the amount of F‐actins (residual F‐actins) in the “OFF” state in response to the cyclic adhesion. Interestingly, for *T* = 1 h, the residual F‐actins within cells increased gradually as the number of cycles increased from 1 to 3 (Figure S21, Supporting Information). However, for *T* = 6 h, the residual F‐actins within cells in the “OFF” state were analogous to that of the static state and were unaffected by the cyclic adhesion (Figure S21, Supporting Information). Together, these results confirm the rationality of the above hypothesis.

The model predicted that cyclic integrin‐mediated adhesion would increase the directional bias of F‐actin in the actin cap after three cycles of loading (for *T* = 1 h followed by 12 h of static culture), relative to that observed following 12 h of static culture with no cyclic loading (Figure 2D–F). Experiments using established methods for quantifying actin alignment^[^ ^30^ ^]^ confirmed this prediction (Figure 2E,F, where 90° represents the dominant direction). The model predicted increasing traction stress, nuclear deformation, and YAP nuclear localization with a decreasing cyclic period (Figure 2G–M). Traction force microscopy measurements on cells after static culture or three cycles of adhesion of various periods confirmed this tendency of traction (Figure 2G,H), as expected from previous reports of contractile force increasing with actin cap alignment.^[^ ^31^ ^]^ It is known that the actin cap is directly connected to the nucleus through linkers of nucleoskeleton and cytoskeleton (LINC) complexes, allowing transmitting forces generated by F‐actins to the nucleus.^[^ ^32^ ^]^ We next sought to determine whether cyclic attachment‐detachment altered the nucleus deformation. Visualization and quantification of nuclear flattening (*λ*_n_, nuclear length over its height) demonstrate that the nuclei were flattened after three cycles of attachment‐detachment stimuli at *T* = 1 or 2 h, while negligible change was observed between static and cyclic groups at *T* = 6 h (Figure 2I,J). These observations suggest that extracellular forces generated by more aligned F‐actins triggered by cyclic integrin‐mediated adhesion can be transmitted to the nucleus, thereby resulting in nucleus flattening. The model also predicted a similar trend in the size of focal adhesions with traction force as the cyclic period varied. This was validated by the experiments using vinculin immunostaining (Figure S22A, Supporting Information). Specifically, the adhesion length increased with increasing cyclic period (*T*) in the short cycle, whereas it decreased with increasing cyclic period (*T*) in the relatively long cycle. In contrast, we observed a reverse trend in the actin flow rate (Figure S22B, Supporting Information).

The simulation results indicate that as the cyclic period is shortened below 10 min, the enhancement of mechanotransduction begins to decline (Figure 2K). The traction force reaches its maximum when the cyclic period falls within the range of 10–60 min, corresponding to attachment and detachment time of 5–30 min (Figure 2K). Remarkably, this optimal range aligns well with the characteristic times of actin assembly during cell spreading (≈15 min) rather than the half‐life of F‐actin (≈50 min).^[^ ^33^ ^]^ These findings suggest that the enhancement of mechanotransduction through cyclic adhesion depends on a nuanced equilibrium between F‐actin generation upon attachment and F‐actin degradation upon detachment. Because nuclear deformation and YAP nuclear localization increase with contractility,^[^ ^22a^ ^]^ we expected experiments to confirm model predictions that nuclear flattening (*λ*_n_) and YAP nuclear localization increase with traction stress over three cycles of adhesions, and this was indeed the case (Figure 2L,M).

Nuclear mechanics mediated by cyclic integrin‐mediated adhesions is related to chromatin remodeling, which affects a series of fundamental cellular processes including mRNA transcription, DNA replication, recombination, repair, etc.^[^ ^22b^ ^]^ Biophysical cues—including stiffness, stretch, and topography—can modulate chromatin organization and accessibility and have been implicated in cell‐fate commitment and cellular plasticity.^[^ ^34^ ^]^ The chromatin remodeling is directly linked to mechanical transmission from the actin cytoskeleton to the nucleus, rather than by signaling pathways or transcriptional regulation.^[^ ^35^ ^]^ The actin cytoskeleton exerts forces on the nucleus across the LINC complexes, which plays a key role in nuclear mechanosensing and can signal directly to chromatin via changes in tension.^[^ ^36^ ^]^ Therefore, further studies were employed to ascertain levels of chromatin condensation by analyzing DAPI staining intensity and calculating the chromatin condensation parameter (CCP). Results from this assay showed that the chromatin condensation parameter (CCP) decreased after three cycles of adhesion for *T* = 1 or 2 h (Figure S23, Supporting Information). This indicated that chromatin remodeling in hMSCs is regulated by cell mechanics that are triggered by cyclic integrin‐mediated adhesions. Notably, the relationship between chromatin condensation and cell proliferation is still unclear and needs further study in the future.

Taken together, the integrated model and experiments suggest that cyclic integrin‐mediated adhesion alters hMSCs mechanosensing due to changes in actin dynamics during cyclic adhesion. Cycling adhesion promoted the formation of a more aligned actin cap that increased contractile force and deformed the nucleus, thus facilitating YAP nuclear localization and altering the interpretation of the mechanical microenvironment via a cytoskeleton‐mediated mechanosensitive pathway.

### 2.4 Attachment and Detachment Affect Cells Over Different Timescales

**Figure 3:**
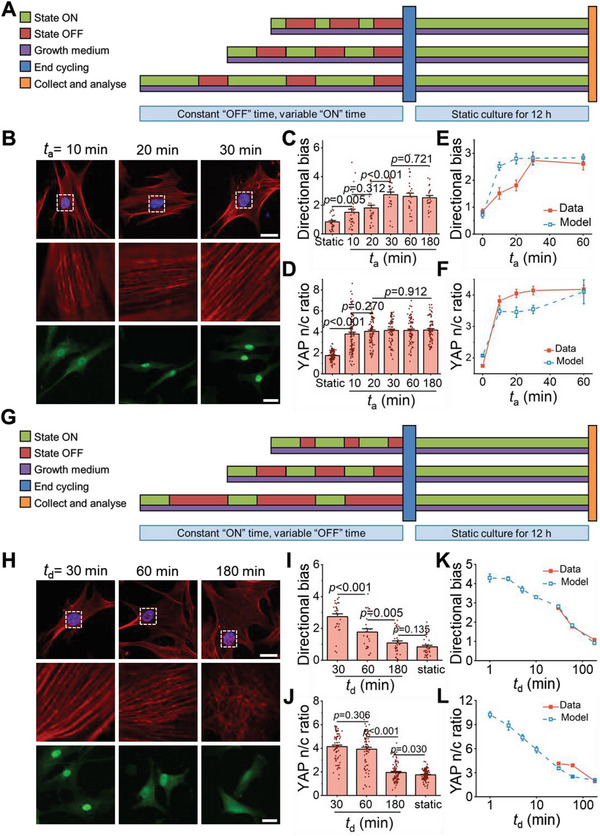
Increasing attachment time promotes F‐actin alignment and YAP nuclear localization, while increasing detachment time does the converse, as the model predicts. A) Schedules of three cycles of adhesion with various attachment times (***t***_**a**_ =  10,  20,  30 **min**) and fixed detachment time ( ***t***_**d**_ =  30 **min**). B) Representative images of F‐actin (red), nuclear (blue), and YAP (green) immunofluorescence in response to cyclic integrin‐mediated adhesion. Scale bar, 20 µm. C) Quantification of ***t***_**a**_‐dependent directional bias of F‐actin (from left to right *n* = 34, 31, 30, 29, 27, and 27 cells, *p* values were obtained using one‐way ANOVA followed by Tukey's post hoc test, error bars are mean ± s.e.m). D) Quantification of ***t***_**a**_‐dependent YAP n/c ratio (from left to right *n* = 112, 113, 92, 75, 77, and 72 cells, *p* values were obtained using one‐way ANOVA followed by Tukey's post hoc test, error bars are mean ± s.e.m). E,F) Comparison of model predictions (blue dashed line) and experimental average values (red solid line) of directional bias of F‐actin (E) and YAP n/c ratio (F) as a function of ***t***_**a**_. G) Schedules of three‐cycle adhesion with varied detachment time (***t***_**d**_) and fixed attachment time ( ***t***_**a**_ =  30 **min**). G) Schedules of three cycles of adhesion with various detachment times (***t***_**d**_ =  30,  60,  180 **min**) and fixed attachment time (***t***_**a**_ =  30 **min**). H) Representative images of F‐actin (red), nuclear (blue), and YAP (green) immunofluorescence in response to cyclic integrin‐mediated adhesion. Scale bar, 20 µm. I) Quantification of ***t***_**d**_‐dependent directional bias of F‐actin (from left to right *n* = 29, 28, 32, and 34 cells, *p* values were obtained using one‐way ANOVA followed by Tukey's post hoc test, error bars are mean ± s.e.m). J) Quantification ***t***_**d**_‐dependent YAP n/c ratio (from left to right: *n* = 75, 79, 86, and 112 cells, *p* values were obtained using one‐way ANOVA followed by Tukey's post hoc test, error bars are mean ± s.e.m). K,L) Comparison of predicted (blue dashed line) and experimental (red solid line) directional bias of F‐actin (K) and YAP n/c ratio (L) as a function of ***t***_**d**_.

When applied to predict how the cyclic attachment and detachment time affect cellular mechanotransduction, the model predicted that detachment time would dominate over attachment time for the cyclic attachment/detachment‐induced cellular mechanotransduction (**Figure** **3**). Experiments with attachment (*t*_a_) and detachment time (*t*_d_) varied independently confirmed these predictions. The model predicted that the directional bias of F‐actin alignment and YAP n/c ratios would both plateau for attachment time exceeding 0.5 h, and experiments confirmed this (Figure 3A–F). Prolonged attachment promoted F‐actin assembly into larger actin bundles, an effect that plateaued within 0.5 h, consistent with previous reports of cell spreading and actin bundle development reaching their fullest extent within 0.5 h.^[^ ^37^ ^]^ For detachment time, the model predicted that the alignment of F‐actin and the YAP n/c ratio monotonically decreased with increasing detachment time in a wide time range, and our experiments confirmed this (Figure 3G–L). The decrease of F‐actin orientation with increasing detachment time suggested an F‐actin disassembly half‐life of ≈1.5 h (Figure 3K), consistent with previous findings.^[^ ^33^ ^]^

The model predicted that detachment time dominated over attachment time in cyclic attachment/detachment‐induced cellular mechanotransduction, with F‐actin directional bias and YAP n/c ratios decreasing over prolonged detachment times. The model predicted that memory of previous cyclic adhesion was lost when detachment time was sufficiently long (more than 3 h) because F‐actin fragments completely depolymerized. This occurred because F‐actin degraded with a time constant on the order of 1.5 h,^[^ ^33^ ^]^ while the effects of prolonged attachment saturated within 0.5 h.^[^ ^38^ ^]^

### 2.5 Molecular Motor‐Clutch Elements Dictate Cell Responses to Cyclic Integrin‐Mediated Adhesion

**Figure 4:**
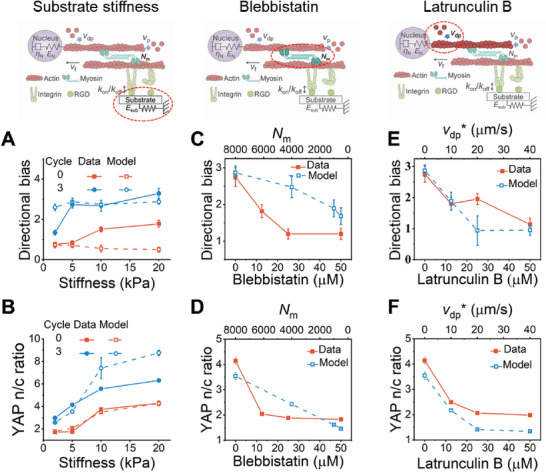
The model predicts the effects of inhibiting the individual components of the molecular motor‐clutch apparatus. A,B) Comparison of experimental (red solid line) and predicted (blue dashed line) directional bias of F‐actin alignment (A) and YAP n/c ratio (B) of cells after static or three cycles of adhesion on substrates of defined stiffness (2, 5, 10, and 20 kPa). C,D) Comparison of experimental (red solid line) and predicted (blue dashed line) directional bias of F‐actin alignment (C) and YAP n/c ratio (D) of cells after static or three cycles of adhesion with prescribed doses of the myosin inhibitor blebbistatin. E,F) Comparison of experimental (red solid line) and predicted (blue dashed line) directional bias of F‐actin alignment (E) and YAP n/c ratio (F) of cells after static or three cycles of adhesion with prescribed doses of the G‐actin sequestering drug latrunculin (B). In all the experiments and simulations, ***t***_**a**_ = 30**min**, ***t***_**d**_ = 30**min**.

We next studied how the individual elements of the motor‐clutch apparatus at focal adhesions, including substrate stiffness, actomyosin contractility, and actin depolymerization, on cell responses to cyclic integrin‐mediated adhesion (**Figure** **4**). We hypothesized that cyclic adhesion would dominate over the effects of substrate stiffness to promote mechanosensing through the effects of accelerated repolymerization dynamics of F‐actin fragments. On static substrates, increasing ECM stiffness (2, 5, 10, and 20 kPa) increased actomyosin contractility and alignment of F‐actin.^[^ ^1^, ^23^ ^]^ However, our model predicted that when *E*_sub_ ≥ 5 kPa, the effects of substrate stiffness would be overridden upon three cycles of adhesion due to the accelerated formation of stress fibers resulting from the cumulative effects of F‐actin dynamics. These effects promoted F‐actin alignment (Figure 4A), and stress‐driven nuclear translocation of YAP (Figure 4B). These predictions were verified by experimental observations (Figure 4A,B; Figure S24, Supporting Information). Note that some discrepancies between model predictions and experimental results were evident in static loading and cases with very low modulus because static effects of stiffness on F‐actin assembly^[^ ^39^ ^]^ were omitted by the dynamic model.

We next hypothesized that actomyosin contractility, which promotes actin polymerization and inhibits actin depolymerization,^[^ ^1^, ^23^, ^40^ ^]^ would increase the availability and length of residual F‐actin fragments that contribute to memory effects in cyclic loading. Our model predicted this: in simulations where contractility was reduced by reducing the number *N*_m_ of myosin motors (Figure 4C), F‐actin alignment and YAP nuclear localization decreased (Figure 4D). Experiments performed in the presence of prescribed concentrations of the myosin inhibitor blebbistatin verified these predicted trends and supported our hypothesis (Figure 4C,D; Figure S25, Supporting Information).

Based on these results, we further hypothesized that restricting actin polymerization directly would also inhibit the effects of cyclic adhesion on F‐actin dynamics and mechanosensing. This effect was predicted by our model in simulations where the F‐actin depolymerization rate ($v_{\mathrm{dp}}^{*}$) was increased: directional bias decreased with increasing F‐actin depolymerization rate, especially as aligned F‐actin began to depolymerize faster than its polymerized (Figure 4E). YAP nuclear localization shows a similar trend (Figure 4F). This prediction was verified and our hypothesis was supported by experiments in which actin polymerization was inhibited using the G‐actin sequestering drug latrunculin B (LatB),^[^ ^28^, ^41^ ^]^ which inhibited the formation of bundled F‐actin, reduced nuclear deformation, and thereby lowered the YAP n/c ratio (Figure 4E,F; Figures S26 and S27, Supporting Information).

Taken together, results support the hypothesis that actin stores memory of previous integrin ligation in the form of F‐actin fragments that persist for a few hours after cytoskeletal depolymerization. This memory is so strong that cyclic integrin‐mediated adhesion can dominate over static cues related to substrate stiffness^[^ ^6^, ^21^, ^42^ ^]^ in the activation of cellular mechanotransduction. Cyclic integrin‐mediated adhesions have a positive effect on mechanotransduction, but there is no report that activated mechanotransduction has an adverse effect on cell function at present. Therefore, it is uncertain that cells are subjected to constant stress that is detrimental to other cellular functions. Importantly, our material system enables the characterization of how cells sense and remember cyclic integrin‐mediated adhesion, and how such cyclic adhesion aligns F‐actin networks and activates transcriptional regulation via YAP. The cyclic system and these observations may have implications for a range of physiological and pathological processes that are regulated by mechanical factors and ECM characteristics.

## 3 Conclusion

We reported a DNA‐mediated molecular system capable of cyclic regulation of integrin‐mediated adhesion. Using this system, we discovered that cyclic adhesion alters intrinsic force‐sensing by hMSCs and thus regulates mechanosensing. Specifically, adhesion with shorter cyclic periods promoted F‐actin alignment, independent of substrate stiffness, and activated cellular mechanotransduction. These effects were consistent with a motor‐clutch‐based model of actin dynamics that accounted for how fragments of F‐actin accelerate subsequent cytoskeletal reassembly and alignment following the resumption of ligation. These persist for a few hours after loss of ligation. Changes to mechanotransduction were consistent with models of aligned F‐actin flattening of the nucleus and promoting YAP nuclear translocation. These effects accumulate with subsequent cyclic adhesion, up to a threshold. These findings reveal that cyclic integrin‐mediated adhesion can alter hMSCs' perception of exogenous biophysical inputs such as ECM stiffness, and thereby regulate their mechanosensing and fate commitment. The cyclic integrin‐mediated adhesion will not only help evolve more relevant culture systems, especially for stem cells, but also improve our understanding of how cyclic mechanical cues regulate cell behavior.

## 4 Experimental Section

### Synthesis of RGD‐DNA Molecules

RGD was integrated into “bioactive strand” via copper‐free click reaction between the cyclooctyne on RGD and the azide on “bioactive strand” according to the procedure shown in Figure S1C (Supporting Information). Briefly, an equal molar ratio of azide‐modified DNA and DBCO‐modified RGD were dissolved in phosphate‐buffered saline (PBS) for 24 h at room temperature to generate the RGD‐DNA molecules. All oligonucleotides were supplied by Sangon Biotech and were listed in Table S1 (Supporting Information). RGD peptides (GRGDSPK) were purchased from Top‐peptide Bio Co, Ltd.

### RGD‐Modified PEG Hydrogel Preparation

PEG hydrogels were prepared by mixing 8‐arm PEG maleimide (PEG‐MAL, 10 kDa, JenKem Technology) and 8‐arm PEG thiol (PEG‐SH, 10 kDa, JenKem Technology) for 30 min at room temperature. Following three washes with PBS, the PEG hydrogels were then reacted with SH‐“immobilizing strand” in PBS and incubated for 1 h at room temperature to form the “OFF” substrate. Next, the hydrogels (“OFF” state) were incubated with RGD‐DNA molecules for 1 h at room temperature to convert the substrate from the “OFF” state to the “ON” state. To remove the RGD molecules that did not react with bioactive strands, the hydrogels were rinsed thrice with PBS. Finally, the prepared hydrogels were used within the same day of preparation. FAM‐labeled RGD was used to characterize the presence of RGD in hydrogels.

### Mechanical Properties of Hydrogels

A DHR3 shear rheometer (TA Instruments) with a parallel plate geometry was used for rheological testing. The storage modulus *G*′ and loss modulus *G*′′ were measured at 1% strain and a frequency of 1 rad s^−1^. The 14 mm diameter PEG hydrogel samples were tested at a constant temperature of 37 °C. Young's modulus *E* was calculated as follows:

(1) $$E\;=\;2\left(1+\nu \right)\sqrt{G{{}^{\prime }}^{2}+G{}^{\prime }{{}^{\prime }}^{2}}$$

where *ν* = 0.5 for the Poisson ratio of PEG hydrogels.^[^ ^22^, ^43^ ^]^

### Coupling Efficiency of RGD and DNA

The coupling efficiency of the FAM‐labeled RGD‐incorporated DNA (bioactive strand) was determined by fluorescence spectroscopy at 522 nm. After preparation, the RGD‐DNA samples were added to the “OFF” substrates for 2 h. Fluorescence emission of the supernatant was measured to calculate coupling efficiency. The control group consisted of only RGD or RGD mixed with DNA lacking an N_3_ group.

### hMSC Isolation and Culture

Human mesenchymal stem cells (hMSCs) were isolated from human bone marrow provided by commercial sources (Cyagen Biosciences). Briefly, cells were obtained from donors by bone marrow aspiration, then monocyte density centrifugation was performed and selected for adherent culture. Standard analytical methods were used to screen cell growth and differentiation into fat and bone. hMSCs were cultured in growth media (Cyagen, HUXMA‐90011), except as noted. For osteogenic differentiation studies, cells were cultured in an osteogenic medium (Cyagen, HUXMA‐90021) to assay the hMSCs osteogenic differentiation capability. Freshly isolated hMSCs were frozen down in 95% fetal bovine serum and 5% dimethylsulphoxide and marked as P1 hMSCs. P1 hMSCs were expanded in growth media to generate P2 cells, which were used in all the reported experiments in this manuscript.

### Cyclic Attachment‐Detachment of Cells During Culture

Hydrogels were sterilized in 75% (v/v) aqueous ethanol for 1 h followed by three rinses with sterilized PBS. In this study, hMSCs were seeded onto PEG substrates at 5 × 10^3^ cells per cm^2^. Prior to cell seeding, the hydrogels were prewetted with growth media for 30 min. The hMSCs were cultured on the “ON” substrates in growth media in a 24‐well plate for 0.5, 1, and 3 h, respectively. The “ON” state was switched to “OFF” state during the hMSCs culture via the addition of 500 µm of “displacement strand”, and continued to culture for 0.5, 1, and 3 h, respectively. The above steps completed an attachment‐detachment process. To restore the substrate to the “ON” state again, a fresh aliquot of 500 µm of RGD‐DNA was added to the substrates. Finally, the cyclic integrin‐mediated adhesion process could be achieved by gradually adding “displacement strand” and RGD‐DNA. The periods and number of cycles could be adjusted as needed. For inhibitors experiments, cells were first subjected to cyclic adhesion, followed by an exchange of the cell culture medium that contained the respective drugs: latrunculin B (Abcam no. 144 291) and blebbistatin (Abcam no. 120 425). All inhibitors were diluted in DMSO, and DMSO alone was used as a control.

### Cell Viability

For cell viability experiments, media was exchanged with PBS containing 2 µm Calcein‐AM and 4 µm ethidium homodimer‐1 for 10 min at 37 °C. The cells were then rinsed with PBS and imaged with Olympus FV3000 confocal microscope.

### Immunostaining and Quantification

Collected samples were fixed at desired time points using 4% paraformaldehyde for 20 min at room temperature. After thoroughly rinsing with PBS, 0.5% Triton‐X was added for 10 min. Subsequently, PBS was used to rinse the samples, which was followed by 5% w/v BSA passivation for 30 min at room temperature. Without rinsing, the primary antibody was directly added into the samples to target the protein of interest and incubate for 12 h at 4 °C. After three PBS rinses, samples were incubated with fluorescently labeled secondary antibodies for 2 h at room temperature, followed by F‐actin staining using Rhodamine Phalloidin (Invitrogen no. R415) incubated for 30 min. All immunostained samples were embedded in ProLong Gold Antifade Reagent with DAPI (Cell Signaling no. 8961) and then stored at 4 °C until imaging with Olympus FV3000 confocal microscope. The following primary antibodies were used: anti‐YAP (Cell Signaling no. 14 074), anti‐RUNX2 (Cell Signaling no. 12 556), anti‐Lamin A/C (Cell Signaling no. 4777). All primary antibodies were used at a 1:100 dilution, and fluorescently labeled secondary antibodies were used at a 1:500 dilution.

The length and orientation of F‐actin were quantified by Filament Sensor (FS) software.^[^ ^30^ ^]^ The directional bias was defined as^[^ ^44^ ^]^:

(2) $$\mathrm{directional}\;\mathrm{bias}\;=\frac{\sum l_{∥}\;}{\sum l_{⊥}}\;$$

where *l*_∥_ is the length of the F‐actin within the direction range of maximum F‐actin accumulation, *l*_⊥_ is the length of the F‐actin within the direction range perpendicular to *l*_∥_.

For the YAP nucleus‐to‐cytoplasm ratios, the nucleus and cytoplasm were identified by F‐actin and DAPI staining, respectively. And the nuc/cyto ratio *R* for YAP was calculated following a procedure used by others,^[^ ^22^, ^45^ ^]^ in which the ratio of the total fluorescence intensity in the nucleus (*I*_nucleus_) to the total fluorescence in the remainder of the cell, was weighted by the areas of the nucleus and the remainder of the cell:

(3) $$R\;=\frac{\left(I_{\mathrm{nucleus}}/A_{\mathrm{nucleus}}\right)\;}{\left(I_{\mathrm{cell}}-I_{\mathrm{nucleus}}\right)/\left(A_{\mathrm{cell}}-A_{\mathrm{nucleus}}\right)}\;$$

where *A*_nucleus_ is the area of the nucleus as measured by DAPI staining. *A*_cell_ is the overall area of the cell as delineated by F‐actin staining. *I*_cell_ is the total fluorescence intensity in the overall cell. Intensities and areas were measured using Image J. The RUNX2 n/c ratio was calculated following the same method.

To analyze chromatin condensation, a chromatin condensation parameter (CCP) was calculated for each nucleus based on the DAPI staining. CCP was generated using a MATLAB script from the Mauck group in which a gradient‐based Sobel edge detection algorithm was used to measure the edge density for individual nuclei.^[^ ^46^ ^]^

### Traction Measurements

To measure the tractions exerted by hMSCs on the substrates, PEG hydrogels with fluorescent microspheres at 2% v/v (Invitrogen no. F8811) were manufactured on glass‐bottom dishes. For imaging the cells and beads, an Olympus FV3000 confocal microscope with an environmental chamber (37 °C, 5% CO_2_) was used. Brightfield images of cells and fluorescence images of beads were captured before and after cell lysis with the addition of sodium dodecyl sulfate. Then these images were analyzed by a previously published MATLAB script^[^ ^47^ ^]^ to obtain a traction force map and the average traction stress exerted by cells on the underlying substrate.

### Actin Flow Measurements

To measure actin rearward flow, hMSCs were transfected with Lifeact‐GFP. Cells were then plated on substrates to perform various cyclic periods of attachment/detachment and imaged every second for 2 min with the confocal microscope. For each cell, a kymograph was obtained at the cell periphery, and actin speed was measured from the slope of actin features observed in the kymograph.

### Statistical Analysis

Data reported throughout the manuscript were mean ± standard error of the mean (s.e.m.) unless otherwise stated. The number of cells/nuclei counted for each condition came from at least three independent experiments, except where noted, and as indicated in each figure legend. Statistical comparisons were performed with Origin (2020) using a one‐way analysis of variance (ANOVA) with Tukey's post hoc test for comparison of multiple groups. Statistically significant differences between the compared groups were identified by *p*‐values less than 0.05.

## Conflict of Interest

The authors declare no conflict of interest.

## Author Contributions

Z.Z. and H.Z. contributed equally to this work. Z.Z., H.Y.Z., M.L., and G.M.G. designed the study, in consultation with G.Q.Z., L.Z.Z., J.T.F., B.G., Z.W., Y.Z., and T.J.L. Z.Z., H.Y.Z., Z.G.Q., L.Z.Z., J.T.F., L.S., H.Z., R.M., and W.C.Z. performed the experiments, modeled, collected, and analyzed the data. Z.Z., H.Y.Z., M.L., Z.G.Q., G.M.G., T.J.L., J.F.Z., and Z.W. prepared the manuscript. All authors discussed the experiments, modeling, and read, and commented on the manuscript.

## Supporting information

Supporting Information

Supplemental Movie 1

## Data Availability

The data that support the findings of this study are available from the corresponding author upon reasonable request.
