# Supplementary material for: Programmable and Reversible Integrin‐Mediated Cell Adhesion Reveals Hysteresis in Actin Kinetics that Alters Subsequent Mechanotransduction
Source: Adv Sci (Weinh). 2023 Oct 17;10(35):2302421. doi: 10.1002/advs.202302421 (PMC10724447; doi:10.1002/advs.202302421)
Supplement: Supplementary file 1 — Supporting Information [file ADVS-10-2302421-s001.pdf]

ADVANCED SCIENCE
Open Access

Supporting Information

for *Adv. Sci.*, DOI 10.1002/advs.202302421

Programmable and Reversible Integrin-Mediated Cell Adhesion Reveals Hysteresis in Actin Kinetics that Alters Subsequent Mechanotransduction

*Zheng Zhang, Hongyuan Zhu, Guoqing Zhao, Yunyi Miao, Lingzhu Zhao, Jinteng Feng, Huan Zhang, Run Miao, Lin Sun, Bin Gao, Wencheng Zhang, Zheng Wang, Jianfang Zhang, Ying Zhang, Hui Guo, Feng Xu, Tian Jian Lu, Guy M. Genin and Min Lin**

Supporting information for "Programmable and reversible integrin-mediated cell adhesion reveals hysteresis in actin kinetics that alters subsequent mechanotransduction"

Zheng Zhang[1,2*], Hongyuan Zhu[1,2*], Guoqing Zhao[1,2], Yunyi Miao[1,2], Lingzhu Zhao[1,2], Jinteng Feng[3], Huan Zhang[1,2], Run Miao[1,2], Lin Sun[1,2], Bin Gao[4], Wencheng Zhang[4], Zheng Wang[5], Jianfang Zhang[6], Ying Zhang[7], Hui Guo[3], Feng Xu[1,2], Tian Jian Lu[8], Guy M. Genin[1,2,9,10], Min Lin[1,2#]

1 The Key Laboratory of Biomedical Information Engineering of Ministry of Education, School of Life Science and Technology, Xi'an Jiaotong University, Xi'an 710049, P.R. China
2 Bioinspired Engineering and Biomechanics Center (BEBC), Xi'an Jiaotong University, Xi'an 710049, P.R. China
3 Department of Medical Oncology, First Affiliated Hospital of Xi'an Jiaotong University, Xi'an 710061, P.R. China
4 Department of Endocrinology, Second Affiliated Hospital of Air Force Military Medical University, Xi'an 710038, P.R. China
5 Department of Hepatobiliary Surgery, First Affiliated Hospital of Xi'an Jiaotong University, Xi'an 710061, P.R. China
6 Department of Gynaecology and Obstetrics Xijing Hospital Fourth Military Medical University, Xi'an 710054, P.R. China
7 Xijing 986 Hospital Department, Fourth Military Medical University, Xi'an 710054, P.R. China.
8 State Key Laboratory of Mechanics and Control of Mechanical Structures, Nanjing University of Aeronautics and Astronautics, Nanjing 210016, P.R. China
9 Department of Mechanical Engineering & Materials Science, Washington University in St. Louis, St. Louis 63130, MO, USA
10 NSF Science and Technology Center for Engineering Mechanobiology, Washington University in St. Louis, St. Louis 63130, MO, USA

* These authors contributed equally to this work
# Corresponding author: minlin@xjtu.edu.cn

This file includes:

Supporting Methods

Method 1: A DNA-driven cell culture platform to achieve cyclic integrin-mediated adhesions.
Method 2: Implementation of a motor clutch-based actin dynamics model.

Supporting Figures

Figure S1. Synthesis of the PEG hydrogel and RGD-DNA molecule.
Figure S2. Cell adhesion on the RGD-DNA modified surfaces.
Figure S3. Determination of the coupling efficiency of FAM-labeled RGD and DNA by fluorescence spectroscopy.
Figure S4. Young's moduli of the PEG hydrogels.
Figure S5. Effect of RGD concentrations on hMSCs spreading.
Figure S6. Removal of RGD from the substrate and the kinetics of RGD conjugation and release.
Figure S7. Kinetics of RGD conjugation and release.
Figure S8. Cyclic presentation of RGD on the substrate.
Figure S9. Cyclic integrin-mediated adhesion.
Figure S10. Cell survival assays after experiencing multiple cycles.
Figure S11. Effect of the presentation of cells on RGD release.
Figure S12. Effect of the number of cyclic adhesion on ECM secretion by cells.
Figure S13. Effect of the number of cyclic adhesion on RGD conjugation in the presence of cells.
Figure S14. Effect of cell presentation on FAM-RGD conjugation.
Figure S15. YAP n/c ratios in hMSCs on the continuous "ON" substrate with different time.
Figure S16. Cyclic integrin-mediated adhesion regulates nuclear localization of RUNX2.
Figure S17. Cyclic integrin-mediated adhesion regulates hMSCs spreading.
Figure S18. Effect of different integrin binding ligands (GFOGER) on cyclic integrin-mediated adhesion.
Figure S19. Effect of different integrin binding ligands (IKVAV) on cyclic integrin-mediated adhesion.
Figure S20. Representative immunofluorescence images of F-actin (red), nuclear (blue) and YAP (green) in response to cyclic integrin-mediated adhesion for 3, 4, 5 cycles for T = 1 h.
Figure S21. Residual F-actin within cells in the "OFF" state versus number and period of cycles.
Figure S22. Cyclic integrin-mediated adhesion regulates the size of focal adhesions and actin flow rate.
Figure S23. Cyclic integrin-mediated adhesion regulates chromatin condensation.
Figure S24. Increasing stiffness promotes F-actin alignment and YAP nuclear localization as predicted by the model.
Figure S25. Decreasing myosin contractility inhibits F-actin alignment and YAP nuclear localization as predicted by the model.
Figure S26. Increasing actin depolymerization rate inhibits F-actin alignment and YAP nuclear localization as predicted by the model.

Figure S27. Inhibiting formation of F-actin reverses cyclic adhesion-enhancement of nuclear deformation.

Supporting Movie

Movie 1: Cyclic integrin-mediated adhesion. Live imaging of hMSCs on substrates during an “ON”-“OFF” cycle (“ON” time for 1 h, “OFF” time for 0.5 h). Scale bar, 20 μm.

Supporting Tables

Table S1: Sequences of all DNA strands used.
Table S2: List of input parameter for CADS.

Supporting References

Supporting Method 1: A DNA-driven cell culture platform to achieve cyclic integrin-mediated adhesions.

The platform is based on a hydrogel (diameter: ~14 mm, thickness: ~200 μm) synthesized by Michael addition between 8-arm PEG maleimide (PEG-MAL) and 8-arm PEG thiol (PEG-SH). A thiol functionalized single stranded DNA (ssDNA), termed the "immobilizing strand", was conjugated to the hydrogel by Michael addition with the unreacted maleimide in the hydrogel (Figure S1A, Supporting Information), which was verified by TAMRA-labeled immobilizing strand (Figure S1B, Supporting Information). To promote cell adhesion, we attached the RGD peptide to a ssDNA (referred to as "bioactive strand") using copper-free click chemistry (Figure S1C, Supporting Information). The resulting RGD-DNA was then hybridized to the complementary immobilizing strand covalently linked to the PEG hydrogels (Figure S2A, Supporting Information), which was verified by FAM-labeled RGD peptides (Figure S2Bi, Supporting Information). The coupling efficiency of RGD and DNA was determined to exceed 90% (Figure S3, Supporting Information).

Substrate with only immobilizing strands were define to the "OFF" state, while substrates conjugated with RGD were define to the "ON" state. Cells plated on the "ON" substrates showed a three-fold increase in cell number relative to cells plated on the "OFF" substrates (Figure S2Bii, C, Supporting Information). Cells plated on the "ON" substrates formed obvious integrin-mediated focal adhesion (indicated by paxillin immunostaining in Figure S2Biii). The cell adhesion is highly specific for RGD, as cell numbers on the substrates modified with double stranded DNA (dsDNA) lacking RGD, RGD conjugated to a scrambled bioactive strand (scrDNA), or the non-bioactive peptide RGE are all as low as that adhered to the "OFF" substrate (Figure S2C, Supporting Information). Moreover, the cell area on these substrates showed a similar trend with cell number (Figure S2D, Supporting Information). The measured Young's moduli of hydrogels showed no significant difference between 'OFF' and 'ON' states (Figure S4, Supporting Information), excluding the effect of substrate stiffness on cell adhesion and spreading. For both "ON" and "OFF" states, the substrates kept the high cell viability (over 90%) for 3 d of culture (Figure S2Biv, E, Supporting Information), confirming their cytocompatibility.

To remove RGD from the "ON" substrates, we introduced a short single-stranded sequence (referred to as "toehold") into the bioactive strand. Adding a fully complementary DNA trigger (referred to as "displacement strand") to the "ON" substrates enables its removal from

the surface by the toehold-mediated strand displacement reaction to switch the substrate back to the "OFF" state, as evident by the dissociation of FAM-labeled RGD from the substrates (Figure S6, Supporting Information). Notably, this process regenerates the immobilizing strand, allowing for a second addition of the RGD-DNA to turn the substrate to the "ON" state. This process can be repeated for multiple cycles (Figure S8, Supporting Information). Thus, the platform could be reversibly switched between "ON" and "OFF" states to favor and disrupt hMSCs adhesion. All the sequences of oligonucleotides used in this work can be found in Table S1.

Supporting Method 2: Implementation of clutch-based actin dynamic model.

Model description and basic assumptions

The clutch-based actin dynamic model is based on the existing motor-clutch model[1] and cell migration simulator[2]. In the model, the cell is composed of several F-actin modules, which emerge one by one depending on the existing G-actin pool[2]. In each module, an F-actin is connected to the substrate by "clutches" (integrin-mediated adhesion). One end of the F-actin is pulled by amounts of "motors" (ATP-dependent myosin II) and away from the integrin clutches, meanwhile the other end of the F-actin polymerizes to extend its length[1]. Based on previous reports on F-actin formation, we assume that: During attachment, (1) the F-actin initiate one-by-one with a random orientation and a fixed initial length[3]. (2) The length of the existing F-actin changes with force-dependent polymerization and depolymeization rates[4]. (3) During detachment, the F-actin degrade gradually with increasing detachment time[3a]. (4) After one cycle of adhesion, the residual F-actin from the last cycle would bundle and align to form the first batch of F-actin, and the orientations of which are opposite on a line to reach a force balance between motor-clutch modules and a central cell body[2]. Then, (1) if the detachment time is long enough and residual F-actin have almost degraded, the first batch of F-actin are short and weak, and the week force generated by which could not sustained its existence, thus the orientation distribution of F-actin is random in the cell. (2) While if the cycle period is short and the residual F-actin are still long enough, the first batch of F-actin are long and strong to dominate in the cell, then the orientation distribution of F-actin is ordered in the cell[5]. Overall, during cyclic adhesion processes, residual F-actin accumulate in each cycle. The difference in length and number of initial residual F-actin leads to distinct final F-actin alignments and the subsequent traction forces in a single cell. For short detachment time, the partially degraded F-actin (residual F-actin) preferentially bundle to and then generate a unidirectionally oriented network of F-actin formed within the cell in the next attachment[5].

Governing equations

(1) Force and motility exerted by a single F-actin module

A single F-actin module a motor-clutch machinery that is composed of n_c integrin clutches, n_m activated myosin motors and F-actin with a length of l_f. The F-actin connects to substrate through bonded integrin clutches in the module. Each integrin clutch could switch between binding and unbinding states at rates of k_on and $k_{\mathrm{off},i}$ respectively. The $k_{\mathrm{off},i}$ of ith clutch in the module could be calculated by[1-2]:

$$k_{\mathrm{off},i} = k_\mathrm{off}^{*} \exp\left(\frac{F_{\mathrm{c},i}}{F_\mathrm{b}}\right) \tag{1}$$

where k_off^{*} is the unloaded clutch unbinding rate, $F_{\mathrm{c},i}$ is the force exerted on the clutch by F-actin, F_b is the characteristic bond rupture force of a clutch.

A single F-actin module exerts a unidirectional traction force to the substrate. The deformations of clutches and the compliant substrate are described as Hookean springs with spring constants of κ_c and κ_s respectively. κ_s could be calculated from Young's modulus of hydrogel according to previous work[1]. The traction force exerting to substrate (F_trac) and the retrograde velocity of F-actin (v_f) are obtained from two main relationships: one is force-velocity relationship as[2]:

$$v_\mathrm{f} = v_\mathrm{f}^{*}\left(1 - \frac{\kappa_\mathrm{s} x_\mathrm{s}}{n_\mathrm{m} F_\mathrm{m}}\right) \tag{2}$$

where v_f^{*} is the unloaded motor velocity, F_m is the stall force for a single myosin motor. The other is the elastic force balance between the clutch and substrate as[2]:

$$F_\mathrm{trac} = \kappa_\mathrm{c} \sum_{i=1}^{n_\mathrm{c}} x_{\mathrm{c},i} = \kappa_\mathrm{s} x_\mathrm{s} \tag{3}$$

where $x_{\mathrm{c},i}$ is the displacement of the ith clutch, x_s is the displacement of the substrate.

It's known that activation level of each F-actin module is determined by its internal stress[6], which is omitted by the existing motor-clutch models[1, 7]. Here, we established a nonlinear relationship between traction force and length of F-actin by integrating Desphande's bio-chemo-mechanical model[6b, 8] and the motor-clutch model[1-2, 9]. Myosin could be activated and then polymerize into a bipolar filament that drive multiple actin filaments[10]. While force generated by the actomyosin filament facilitates adhesion maturation (clutches) could further

enhance the myosin activation signaling[10a]. Considering traction force-dependent activation of actomyosin[6b, 6c], the activation level of the F-actin module (η) is calculated by a first-order kinetic equation:

$$\frac{d\eta}{dt} = k_{\mathrm{a}}(1-\eta) - k_{\mathrm{d}}\left(1 - \frac{F_{\mathrm{mod}}}{n_{\mathrm{m}}F_{\mathrm{m}}}\right)\eta \tag{4}$$

where k_{a} and k_{d} are rate constants of the activation and deactivation of myosin motors respectively, n_{m0} is the total numbers of myosin motors (including activated and deactivated myosin motors) that attach to the F-actin and $n_{\mathrm{m0}}F_{\mathrm{m}}$ represents the traction force at the maximum activation level. The force transmitted to the force-sensitive elements (*e.g.* formin[4]) on actin filament from clutches was calculated according to its activation level and traction force as:

$$F_{\mathrm{mod}} = \eta F_{\mathrm{trac}} \tag{5}$$

As an F-actin module is created, the numbers of myosin motors and clutches are assigned according to the length of the F-actin[2]:

$$n_{\mathrm{m}} = n_{\mathrm{m}}^{*}\frac{n_{\mathrm{m,free}}}{n_{\mathrm{m,tot}}}\frac{l_{\mathrm{f}}}{l_{\mathrm{in}}} \tag{6}$$

$$n_{\mathrm{c}} = n_{\mathrm{c}}^{*}\frac{n_{\mathrm{c,free}}}{n_{\mathrm{c,tot}}}\frac{l_{\mathrm{f}}}{l_{\mathrm{in}}} \tag{7}$$

where n_{m}^{*}, n_{c}^{*} are maximum number of motor and clutches assigned to an F-actin module, $n_{\mathrm{m,free}}$, $n_{\mathrm{c,free}}$ are numbers of free myosin motors and clutches, $n_{\mathrm{m,tot}}$, $n_{\mathrm{c,tot}}$ are total numbers of myosin motors and clutches in the cell.

(2) The generation, polymerization, depolymerization, capping and degradation of F-actin modules

In a cell, F-actin modules generate and vanish dynamically. The total number of F-actin modules is denoted as N_{mod}. Modules of initial length l_{in} and initial random direction θ are created at a rate of k_{mod}, which could be calculated according to reported literature[2, 11]:

$$k_{\mathrm{mod}} = k_{\mathrm{mod}}^{*}\left(\frac{A_{\mathrm{G}}}{A_{\mathrm{T}}}\right)^{4} \tag{8}$$

where k_{mod}^{*} is the maximum rate of module formation, A_{G} is the amount of G-actin, A_{T} is the total amount of actin.

During attachment to the substrate, F-actin extend their lengths by polymerizing at a velocity as[2]:

$$v_{\mathrm{p}} = v_{\mathrm{p}}^{*}\left(\frac{A_{\mathrm{G}}}{A_{\mathrm{T}}}\right) \tag{9}$$

where v_{p}^{*} is the maximum F-actin polymerization velocity. On the other hand, F-actin also shorten their length by depolymerizing at the retrograde velocity (v_{f}). It was observed that F-actin disassemble at sufficiently low stresses[12], which could be attributed to the polymerization and depolymerization rates[4] of actin that increased and decreased separately with increasing tensile force exerted on formin (aF_{mod}, a is a scaling factor, corresponding to the fraction of traction force experienced by formin). Based elongation rate and depolymerization rate measured for the individual F-actin[4], the maximum polymerization rate and depolymerization rate of F-actin modules in our model were defined as[4]:

$$v_{\mathrm{p}}^{*} = \frac{k_{\mathrm{on}}^{\mathrm{act}}}{1 + \frac{1 - p_0}{p_0}\exp(-aF_{\mathrm{mod}}\delta/k_{\mathrm{B}}T)} \tag{10}$$

$$v_{\mathrm{dp}} = \frac{k_{\mathrm{off}}^{\mathrm{act}}}{1 + \frac{p_0}{1 - p_0}\exp(aF_{\mathrm{mod}}\delta/k_{\mathrm{B}}T)} + v_{\mathrm{dp}}^{*} \tag{11}$$

where $k_{\mathrm{on}}^{\mathrm{act}}$ is the rate constant of actin subunit adding to the F-actin module. b is a fitting parameter, p_0 is the probability to find the FH2 dimer (a domain of formin, opening which favors elongation of actin) in the open conformation in the absence of force. δ is the actin monomer size. k_{B} is Boltzmann's constant. T is the absolute temperature. $k_{\mathrm{off}}^{\mathrm{act}}$ is the rate constant that actin subunit disassociates from the F-actin module. v_{dp}^{*} is the minimum value of depolymerization.

Modules are capped with at a rate of k_{cap}. A capped module stop polymerization and degrade gradually. When $l_{\mathrm{f}} \leq l_{\mathrm{min}}$, the F-actin module vanishes.

In an F-actin module, which polymerizes at velocity v_{p} and depolymerizes at velocity v_{dp}. Then, after time t, the length of F-actin is defined as:

$$l_{\mathrm{f}}(t) = l_{\mathrm{f}}(0) + \sum_{i=0}^{t}\left(v_{\mathrm{p}} - v_{\mathrm{dp}}\right)dt \tag{12}$$

After capping, the length of the module is

$$l_{\mathrm{f}}(t) = l_{\mathrm{f}}(0) + \sum_{i=0}^{t} -v_{\mathrm{dp}}dt \tag{13}$$

During detachment, all modules vanish and the F-actin begin to degrade gradually with the detachment time (t_d). The length of residual F-actin follow an exponential function as[3a]:

$$l_\mathrm{r} = l_\mathrm{f} \exp(-k_\mathrm{d} t_\mathrm{d}) \tag{14}$$

where k_d is the degradation kinetics constant of F-actin. In the subsequent attachment, residual F-actin will preferentially crosslink parallel to the pair of initial modules with opposite directions[5], with lengths of no more than l_max.

(3) The force balance among F-actin modules and cell body

The cell body is considered as a no motor module, in which $n_\mathrm{c,cell}$ clutches are associated. The cell body is dragged by N_mod F-actin modules and thus exert traction force on substrate (F_cell), which could be expressed as[2]:

$$F_\mathrm{cell} = \sum_{i=1}^{n_\mathrm{c,cell}} \kappa_\mathrm{c} x_{\mathrm{c},i} = \kappa_\mathrm{s} x_\mathrm{s,cell} \tag{15}$$

where $x_\mathrm{s,cell}$ is the substrate displacement provided by cell body. The traction forces on substrate provided by a cell (F_cell) and F-actin modules (F_trac) keep in balance at any time as[2]:

$$F_\mathrm{cell} + \sum_{j=1}^{n_\mathrm{mod}} F_{\mathrm{trac},j} = 0 \tag{16}$$

where $F_{\mathrm{trac},j}$ is the traction force provided by the jth F-actin module.

The joint traction force exerted to substrate (F_sub) have two direction components and could be calculated by as:

$$F_{\mathrm{sub},x} = \left(\sum_{j=1}^{n_\mathrm{mod}} \left| F_{\mathrm{trac},j} \cos(\theta_j) \right| + \left| F_\mathrm{cell} \cos(\theta_\mathrm{cell}) \right| \right) / 2 \tag{17}$$

$$F_{\mathrm{sub},y} = \left(\sum_{j=1}^{n_\mathrm{mod}} \left| F_{\mathrm{trac},j} \sin(\theta_j) \right| + \left| F_\mathrm{cell} \sin(\theta_\mathrm{cell}) \right| \right) / 2 \tag{18}$$

where θ_j is the angle of force provided by the jth F-actin module and θ_cell is the angle of force provided by the cell body. Then the joint traction force exerted to substrate (F_sub) could be calculated by:

$$F_\mathrm{sub} = \sqrt{F_{\mathrm{sub},x}^2 + F_{\mathrm{sub},y}^2} \tag{19}$$

Implementation and algorithm

Similar to the previous work[2], the model algorithm is described below.

During cell attachment:

(1) Initialize a cell with two equally modules of length (l_{in}) while opposite in orientations (θ_0 and $\theta_0 + \pi$) to reach a force balance. If there exist residual F-actin with length of l_{r}, equally assign the lengths of residual F-actin to the two initial modules until their length reaches the maximum value ($l_{\max}$). Equally assign remained unassigned residual F-actin to other new F-actin modules parallel to the two initial modules, while still keep their lengths below $l_{\max}$.

(2) Calculate module birth rate (k_{mod}, eq. 8) and clutch unbinding rates ($k_{\mathrm{off},i}$, eq. 1)

(3) Calculate time to next event (dt).

(4) Determine which event to execute.

(5) Calculate F-actin retrograde flow rate (v_{f}, eq. 2), polymerizing velocity (v_{p}, eq. 9), activation level (η, eq. 4), and traction force (F_{trac}, eq. 3) for each module.

(6) Update length of each F-actin module (l_{f}) by eq. 12 or 13, and update associated numbers of motors (n_{m}) and clutches (n_{c}) by eq. 7.

(7) Update the positions and forces of clutches and substrate for each uncapped F-actin module.

(8) Execute event.

(9) Destroy modules shorter than the minimal length ($l_{\min}$).

(10) Evaluate force balance to determine the position, traction force (F_{cell}), the substrate extension ($x_{\mathrm{s,cell}}$), clutch extensions ($x_{\mathrm{c},i}$) of the cell body.

(11) Return to step 2.

When switches to cell detachment:

(12) Calculate the lengths of residual F-actin (l_{r}) by eq. 14.

(13) Return to step 1.

Supporting Figures

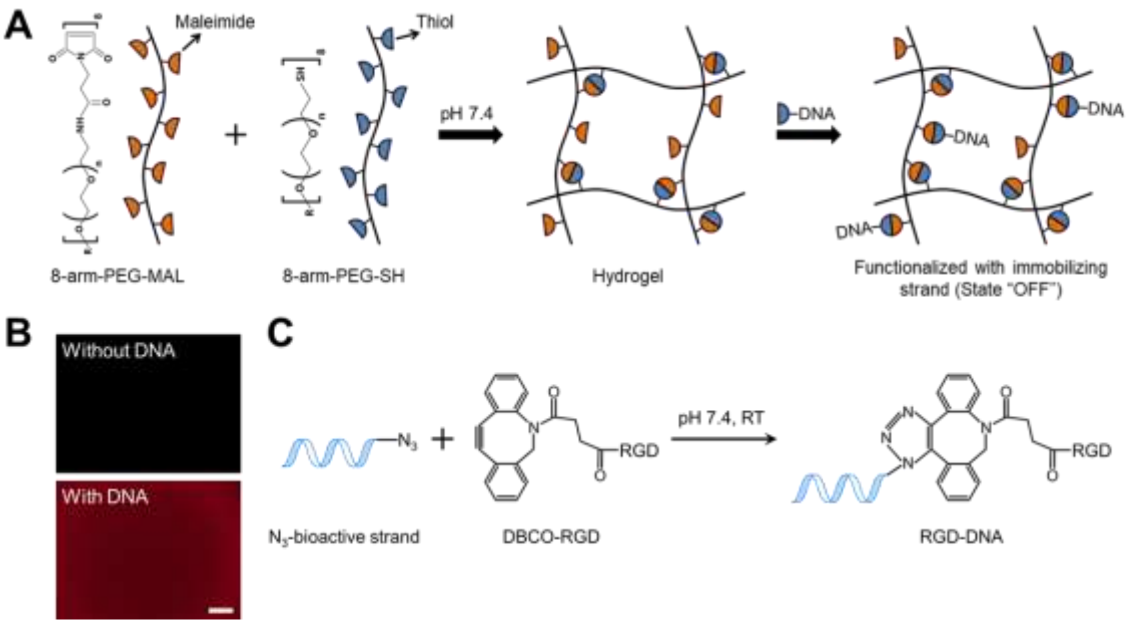

Figure S1. Synthesis of the PEG hydrogel and RGD-DNA molecule. A. Synthetic protocol for coupling immobilizing strand to the PEG hydrogel. The eight-armed PEG maleimide (8-arm-PEG-MA) and eight-armed PEG thiol (8-arm-PEG-SH) was mixed to form a PEG hydrogel by the thiol-Michael addition reaction. SH-functionalized single stranded DNA (ssDNA), termed as the "immobilizing strand", was then conjugated to the PEG hydrogel by reacting with the remaining maleimides. R for hexaglycerol core structure. **B**. Fluorescence imaging of the hydrogels with or without TAMRA-labeled immobilizing strand. Scale bar, 100 µm. **C**. Synthetic scheme for the RGD-DNA molecule. RGD was integrated to DNA (*i.e.*, bioactive strand) via copper-free click reaction between the cyclooctyne on RGD and the azide on bioactive strand.

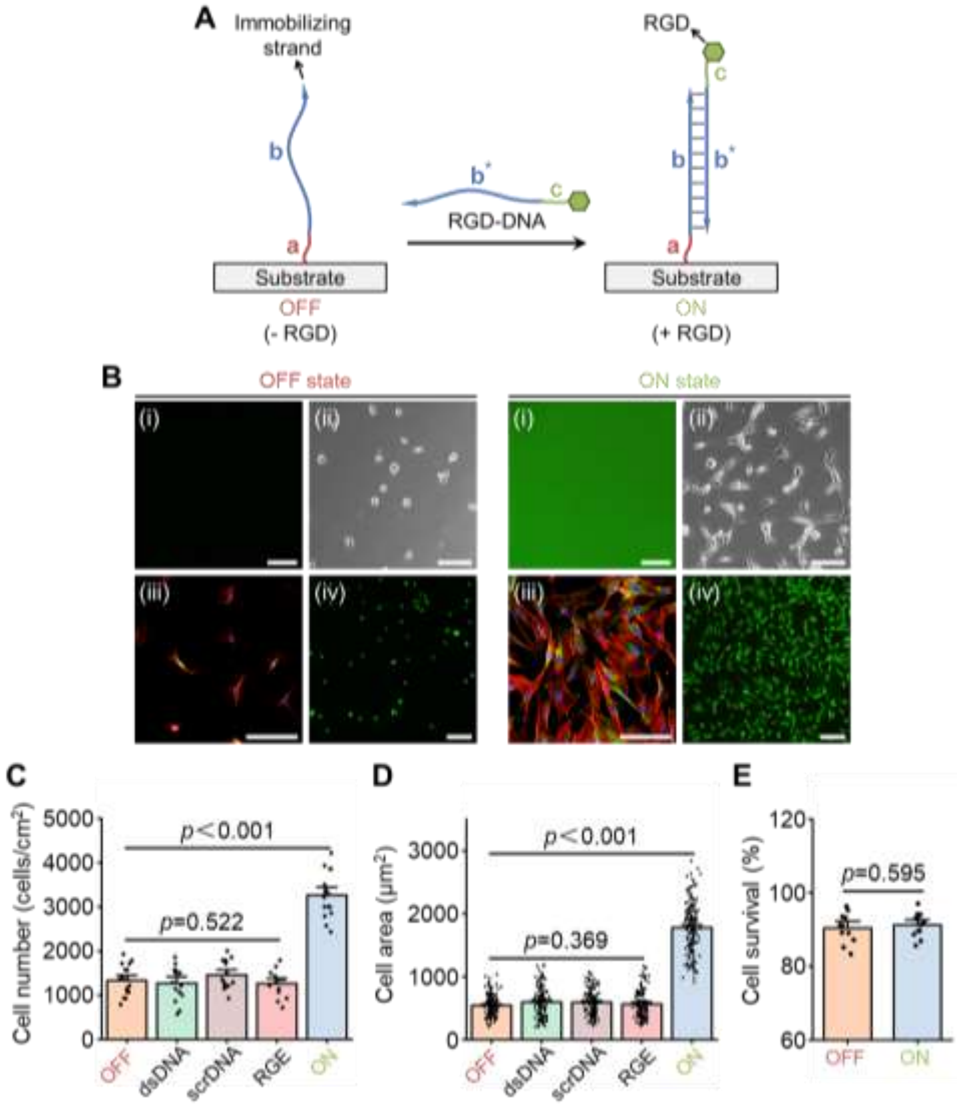

Figure S2. Cell adhesion on the RGD-DNA modified surfaces. A. Schematic illustration for conjugating RGD to the hydrogel via DNA hybridization. **B**. Characterization of RGD immobilization and hMSCs adhesion after 24 h of culture on “OFF” and “ON” substrates. (i) Fluorescence images of FAM-labeled RGD. (ii) bright-field images of cells. (iii) immunofluorescence images of cells (green: paxillin, red: F-actin, blue: DAPI). (iv) live-dead staining images of cells (green: live cells, red: dead cells). Scale bar, 100 μm. **C**. Quantification of cell numbers seeded on various coated substrates (n = 5 samples for each group). **D**. Quantification of cell area on various coated substrates (from left to right: n = 135, 127, 132, 117, 156 cells). **E**. Cell viability on “ON” and “OFF” substrates (from left to right: n = 11, 12 samples). Data are presented as mean ± s.e.m., and p values were obtained using one-way ANOVA followed by Tukey’s post hoc test.

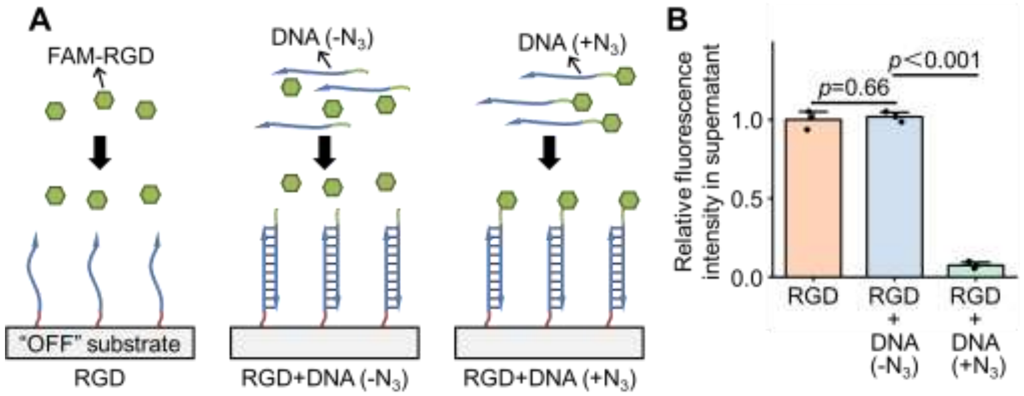

Figure S3. Determination of the coupling efficiency of FAM-labeled RGD and DNA by fluorescence spectroscopy. A. The prepared RGD-DNA molecules were added to the "OFF" substrates and the fluorescence values of the supernatant were detected at 522 nm. DBCO-RGD mixed with N_3-DNA group (RGD+DNA (+N_3)) as the experimental group. Only DBCO-RGD group (RGD) and DBCO-RGD mixed with DNA without the N_3 group (RGD+DNA (-N_3)) as the control group. The coupling efficiencies were calculated through the difference of fluorescence intensities between the RGD+DNA (-N_3) and RGD+DNA (+N_3). **B**. Relative fluorescence intensity in the supernatant for the same conditions in (A) (n = 3 samples per group, p values were obtained using one-way ANOVA followed by Tukey's post hoc test, error bars are mean ± s.e.m).

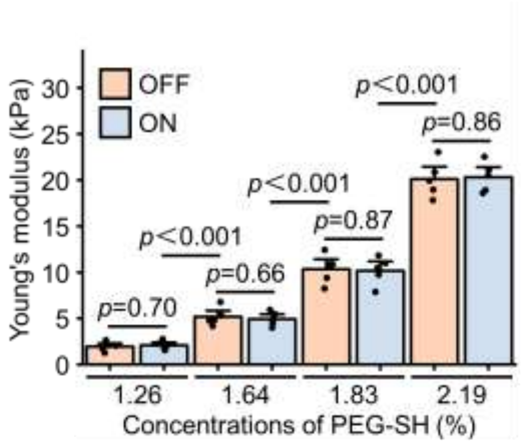

Figure S4. Young's moduli of the PEG hydrogels. Young's moduli of hydrogels synthesized at different concentrations of PEG-SH at "ON" and "OFF" states (n = 5 samples per group, p values were obtained using one-way ANOVA followed by Tukey's post hoc test, error bars are mean ± s.e.m.). The concentration of PEG-MAL was fixed at 5 %. Results indicate that modification of RGD do not alter the stiffness of PEG hydrogels in this system.

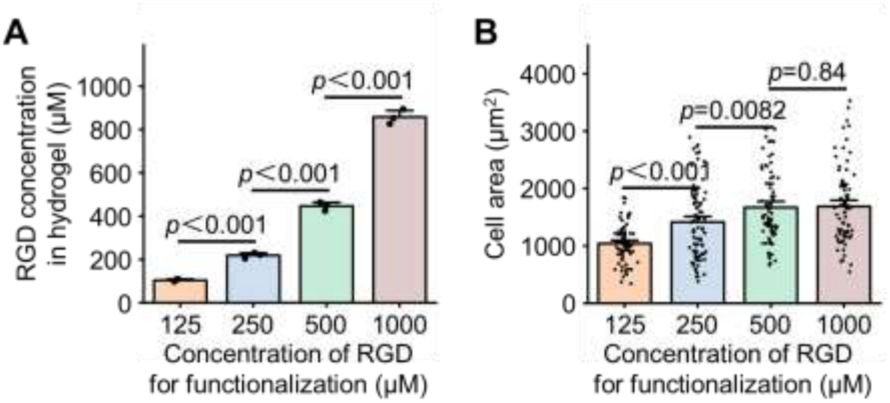

Figure S5. Effect of RGD concentrations on hMSCs spreading. A. The relationship between the amount of RGD used for the functionalization and quantity of peptide attached to the substrates (n = 3 samples per group, p values were obtained using one-way ANOVA followed by Tukey's post hoc test, error bars are mean ± s.e.m). **B**. The relationship between the amount of RGD used for the functionalization and cell area (from left to right: n = 76, 91, 85, 83 cells, p values were obtained using one-way ANOVA followed by Tukey's post hoc test, mean ± s.e.m).

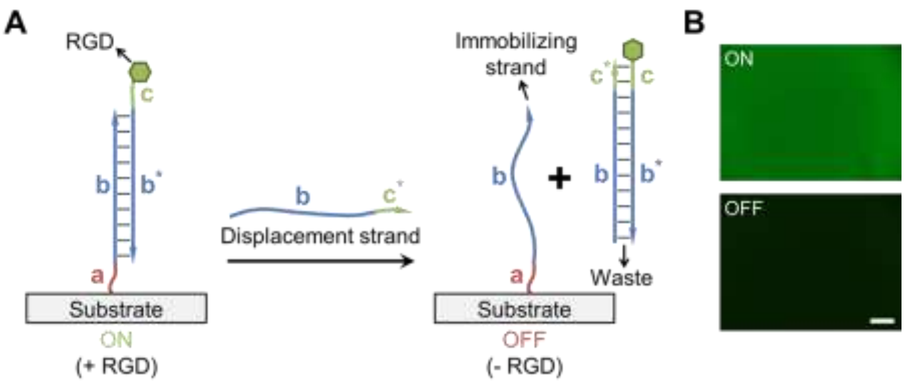

Figure S6. Removal of RGD from the substrate and the kinetics of RGD conjugation and release. A. Schematic illustration of RGD removal via toehold-mediated strand displacement reaction. Engineering a toehold into the "bioactive strand" allows its removal by a fully complementary displacement strand, thereby switching the substrate to "OFF" state. **B**. The presentation (green) and removal (black) of RGD was confirmed using a FAM-labeled RGD-DNA. Adding the FAM-labeled RGD resulted in a fluorescent surface ("ON"), while removal via toehold-mediated strand displacement abolished any fluorescence ("OFF"). Scale bar, 100 μm.

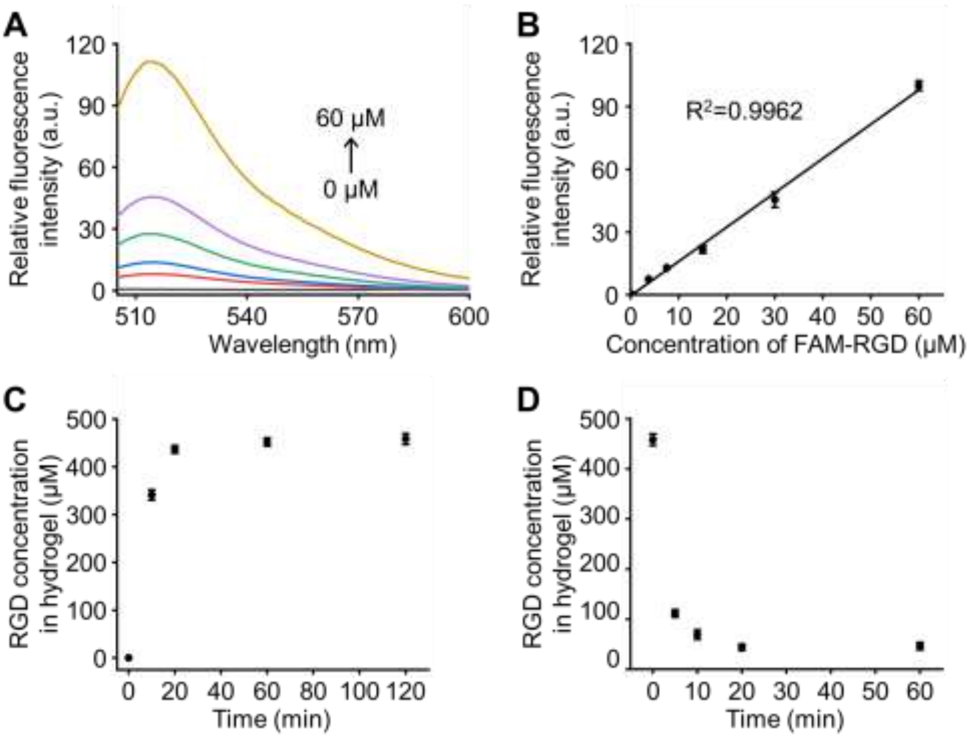

Figure S7. Kinetics of RGD conjugation and release. A-B. Fluorescence spectrum standard curves relating fluorescence signals to the concentration of FAM- RGD in solution. **A**. Fluorescence spectra of the solution with 0, 3.75, 7.5, 15, 30, and 60 μM of FAM-RGD. **B**. Linear regression between fluorescence intensity at 522 nm and concentrations of FAM-RGD (n = 3 samples per group). **C-D**. According to standard curves in B, the conjugation and release kinetics of RGD in the PEG hydrogel could be characterized by measuring the fluorescence intensity of FAM-RGD in the supernatant. **C**. Conjugation kinetics of RGD as characterized after hydrogels were treated with 500 μM FAM-RGD (n = 3 samples per group). **D**. Release kinetics of RGD as characterized after hydrogels functionalized with FAM-RGD were incubated in 500 μM solution of displacement strand (n = 3 samples per group).

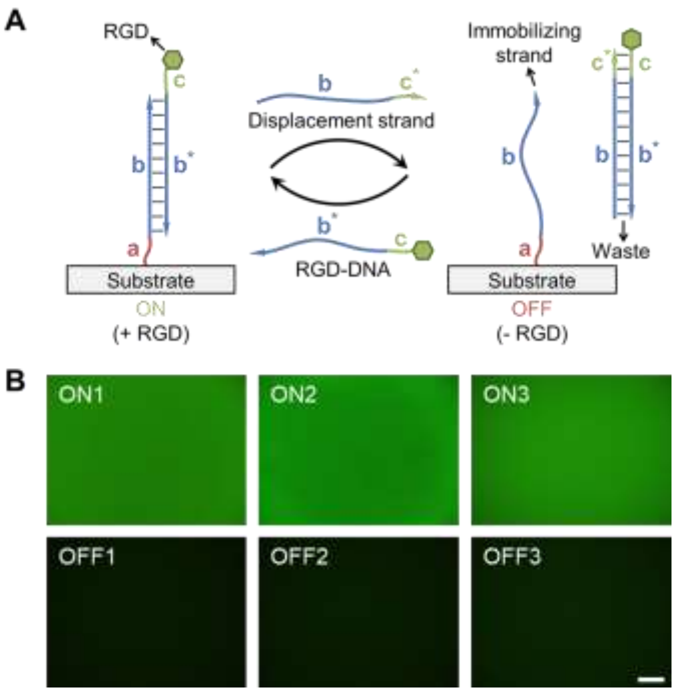

Figure S8. Cyclic presentation of RGD on the substrate. A. Schematic illustration for cyclic presentation of RGD on the substrate by the DNA hybridization and toehold-mediated strand displacement reaction. **B**. The cyclic presentation and removal of RGD were confirmed by a FAM-labeled RGD. Adding the FAM-labeled RGD resulted in a fluorescent surface ("ON1"), and removing it via toehold-mediated strand displacement abolished any fluorescence ("OFF1"). OFF state regenerates the free immobilizing strand, thus allowing the reintroduction of RGD-DNA to restore the "ON" state ("ON2"). This process can be repeated for multiple cycles as indicated by the changes of the fluorescence. Scale bar, 100 µm.

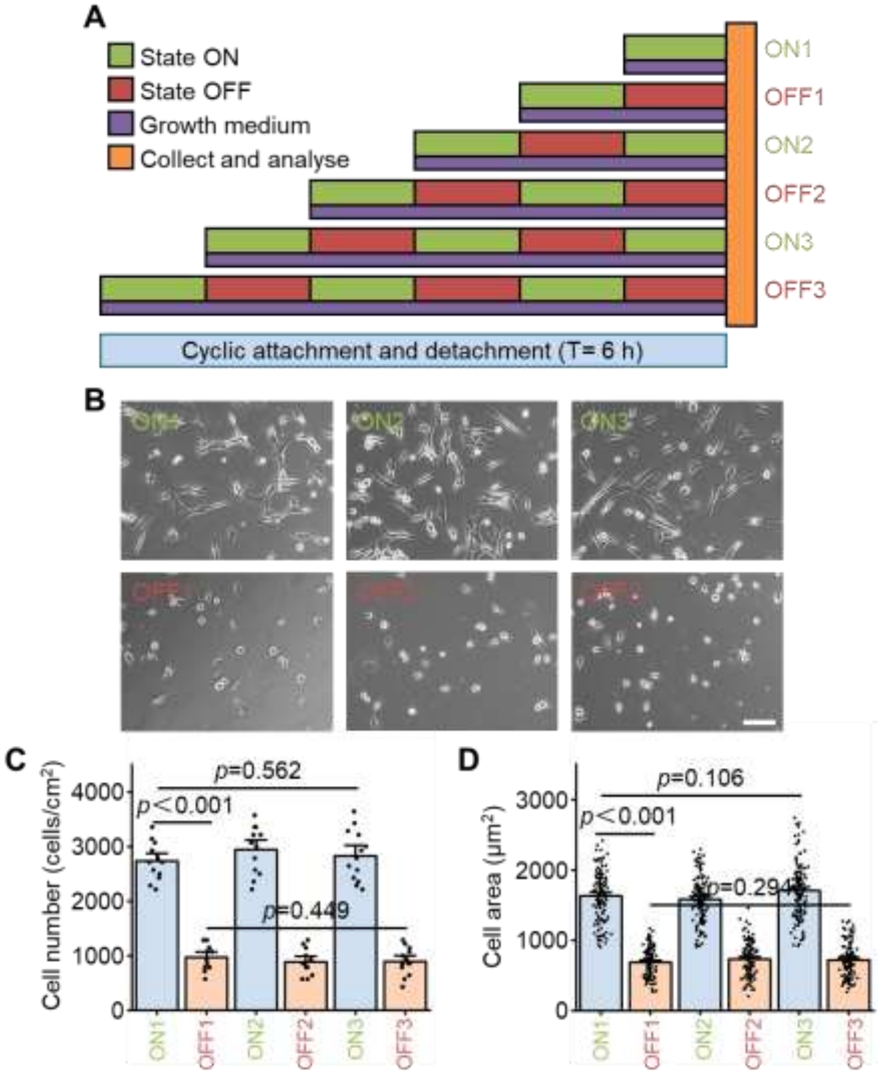

Figure S9. Cyclic integrin-mediated adhesion. A. Dosing of cyclic integrin-mediated adhesion applied to hMSCs cultured on hydrogels in growth media (purple). hMSCs experienced prescribed numbers of "ON" (green)/"OFF" (red) cycles, and then collection and analysis (yellow). At the end of each state, the substrate was gently rinsed, and unbound cells were removed through media exchange. **B**. Representative phase-contrast images of hMSCs seeded on substrates after multiple cycles of switching the substrates between "ON" and "OFF" states (T = 6 h). Scale bar, 100 μm. **C**. Corresponding quantification of cell numbers in the same conditions of (A) (from left to right: n = 13, 12, 13, 12, 13, 13 samples, p values were obtained using one-way ANOVA followed by Tukey's post hoc test, error bars are mean ± s.e.m). **D**. Corresponding quantification of cell area in the same conditions of (A) (from left to right: n = 124, 109, 122, 108, 136, 115 cells, p values were obtained using one-way ANOVA followed by Tukey's post hoc test, error bars are mean ± s.e.m).

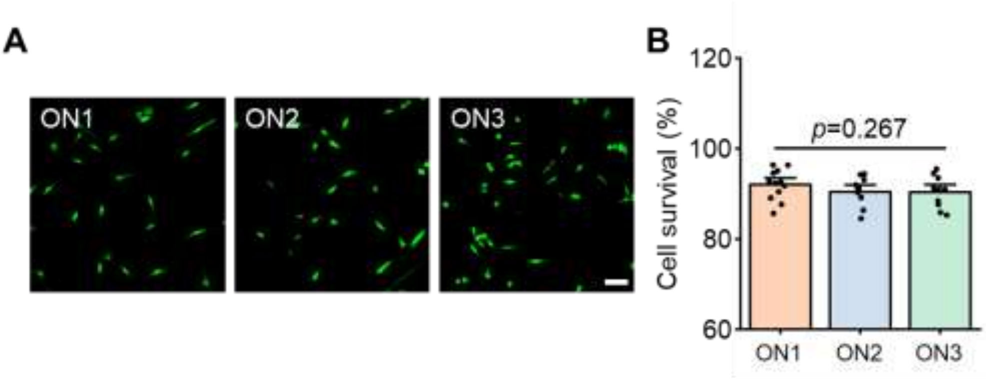

Figure S10. Cell survival assays after experiencing multiple cycles. A. Live-dead fluorescent images of cells (green for live and red for dead). Scale bar, 100 μm. **B**. Corresponding quantification of the cell viability on indicated substrates (from left to right: n = 12, 10, 10 samples, p values were obtained using one-way ANOVA followed by Tukey's post hoc test, error bars are mean ± s.e.m).

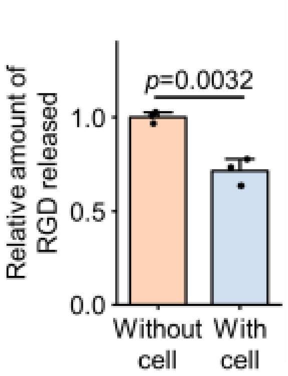

Figure S11. Effect of the presentation of cells on RGD release. The amount of RGD released in the presence or absence of cells on the substrates (n = 3 samples per group, p values were obtained using one-way ANOVA followed by Tukey's post hoc test, mean ± s.e.m).

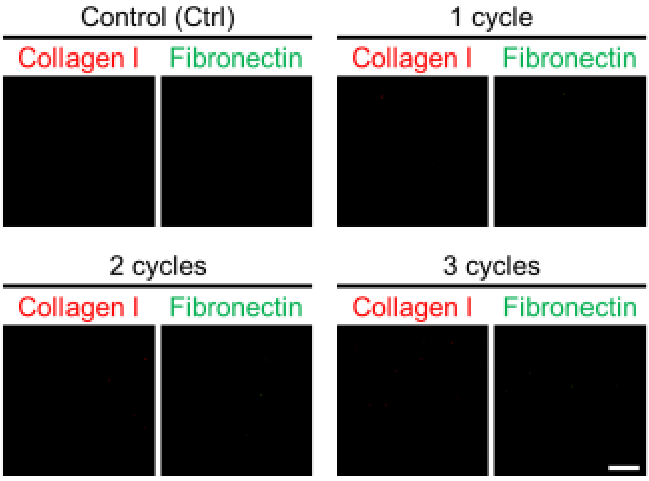

Figure S12. Effect of the number of cyclic adhesion on ECM secretion by cells. Representative immunofluorescence images of collagen I and fibronectin in hMSCs to experience various number (1, 2 or 3) of cyclic adhesion (T = 6 h). The substrates with no cells were control groups (Control, Ctrl). Scale bar, 100 μm.

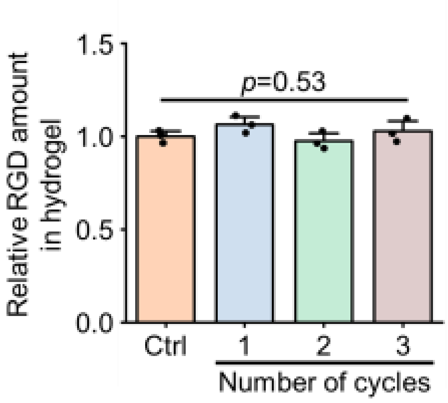

Figure S13. Effect of the number of cyclic adhesion on RGD conjugation in the presence of cells. Relative RGD amount in the presence of cells to experience various number (1, 2 or 3) of cyclic adhesion (T = 6 h). The substrates with no cells were control groups (Ctrl) (n = 3 samples per group, p values were obtained using one-way ANOVA followed by Tukey's post hoc test, error bars are mean ± s.e.m).

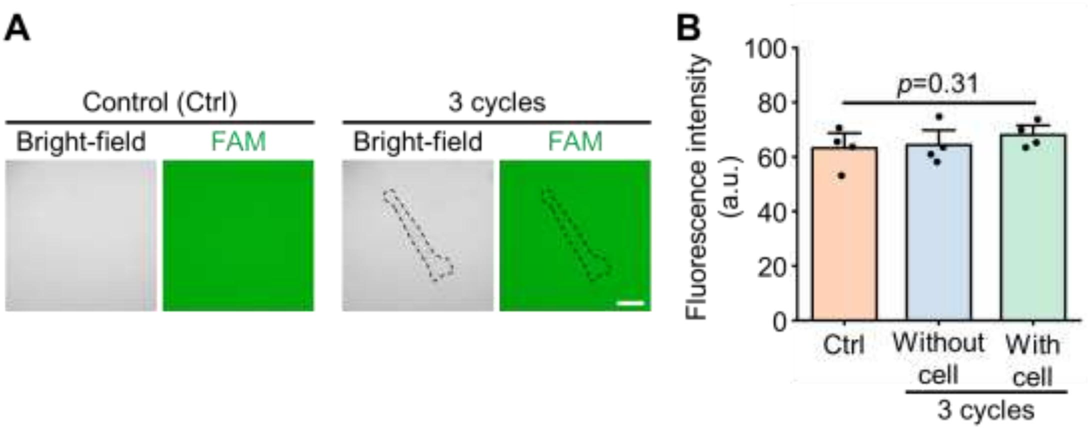

Figure S14. Effect of cell presentation on FAM-RGD conjugation. A. Bright-field and fluorescence images of the "ON" substrate surface with or without hMSCs after 3 cycles adhesion (T = 6 h). The substrates with no cells were control groups (Ctrl). Scale bar: 30 µm. **B**. Corresponding quantification of RGD fluorescence intensity on the substrate surface for the same conditions in (A) (n = 4 samples per group, p values were obtained using one-way ANOVA followed by Tukey's post hoc test, error bars are mean ± s.e.m).

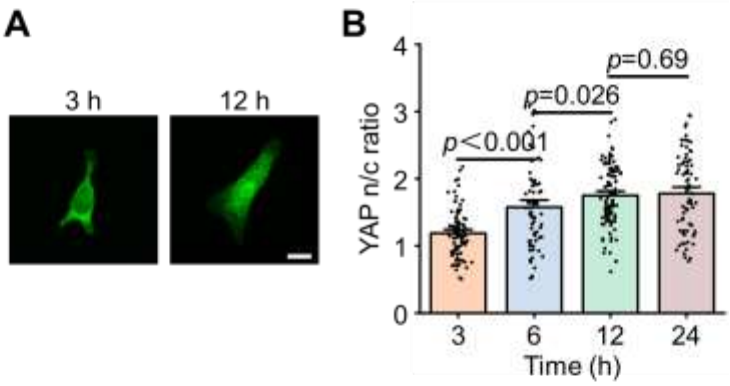

Figure S15. YAP n/c ratios in hMSCs on the continuous "ON" substrate with different time. A. Representative immunofluorescence images of YAP in hMSCs. Scale bar, 20 μm. **B**. Quantification of YAP n/c ratios in response to different attachment time for 3, 6, 12, 24 h (from left to right: n = 87, 74, 112, 81 cells, p values were obtained using one-way ANOVA followed by Tukey's post hoc test, error bars are mean ± s.e.m).

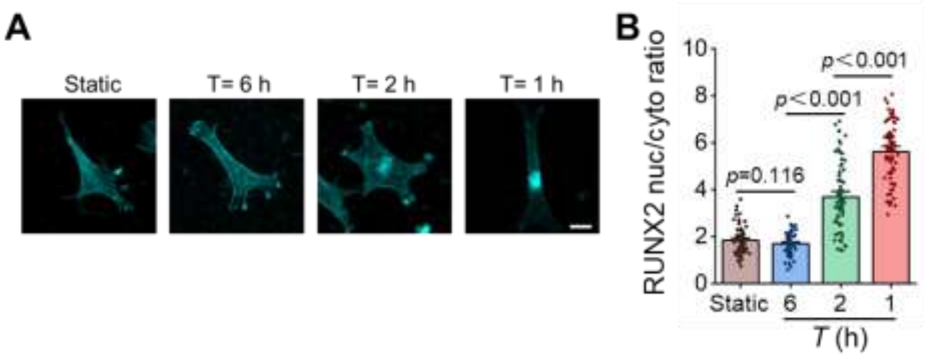

Figure S16. Cyclic integrin-mediated adhesion regulates nuclear localization of RUNX2. **A**. Representative immunofluorescence images of RUNX2 of hMSCs after static or three cycles of adhesions for T = 6, 2, 1 h in osteogenic medium. Scale bar, 20 µm. **B**. Corresponding quantification of RUNX2 n/c ratios for the same conditions in (A) (from left to right: n = 67, 63, 77, 73 cells, p values were obtained using one-way ANOVA followed by Tukey's post hoc test, error bars are mean ± s.e.m).

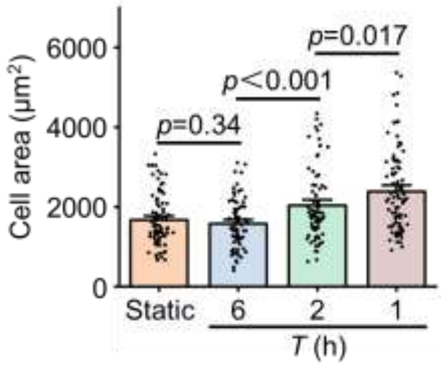

Figure S17. Cyclic integrin-mediated adhesion regulates hMSCs spreading. Quantification of cell area after experiencing static or three cycles of adhesions for T = 6, 2, 1 h (from left to right: n = 85, 77, 82, 92 cells, p values were obtained using one-way ANOVA followed by Tukey's post hoc test, error bars are mean ± s.e.m).

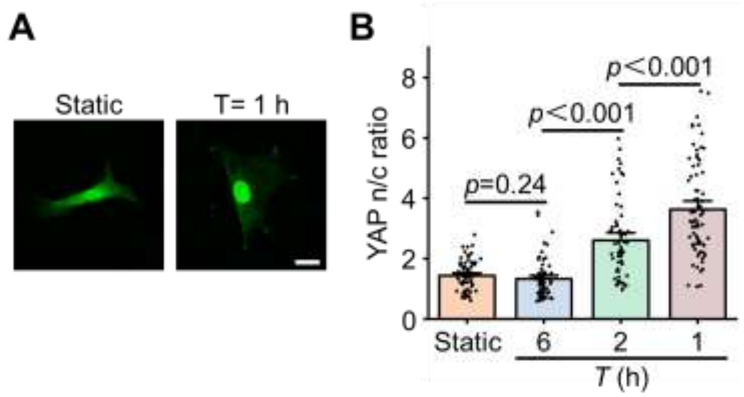

Figure S18. Effect of different integrin binding ligands (GFOGER) on cyclic integrin-mediated adhesion. A. Representative immunofluorescence images of YAP in hMSCs. Scale bar, 20 µm. **B**. Quantification of YAP n/c ratios after static or three cycles of adhesions for T = 6, 2, 1 h with GFOGER instead of RGD (from left to right: n = 77, 68, 61, 73 cells, p values were obtained using one-way ANOVA followed by Tukey's post hoc test, error bars are mean ± s.e.m).

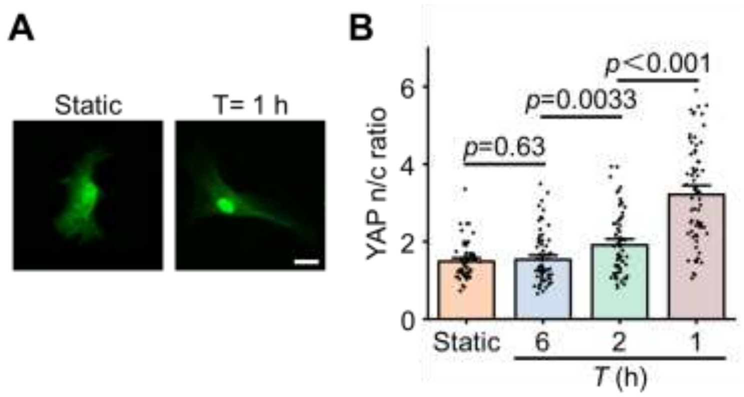

Figure S19. Effect of different integrin binding ligands (IKVAV) on cyclic integrin-mediated adhesion. A. Representative immunofluorescence images of YAP in hMSCs. Scale bar, 20 µm. **B**. Quantification of YAP n/c ratios after static or three cycles of adhesions for T = 6, 2, 1 h with IKVAV instead of RGD (from left to right: n = 62, 73, 65, 68 cells, p values were obtained using one-way ANOVA followed by Tukey's post hoc test, error bars are mean ± s.e.m).

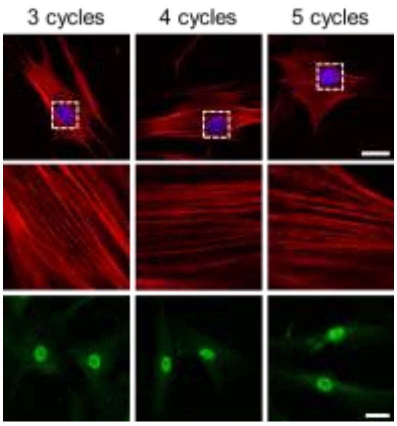

Figure S20. Representative immunofluorescence images of F-actin (red), nuclear (blue) and YAP (green) in response to cyclic integrin-mediated adhesion for 3, 4, 5 cycles for *T* = 1 h. Scale bar, 20 μm.

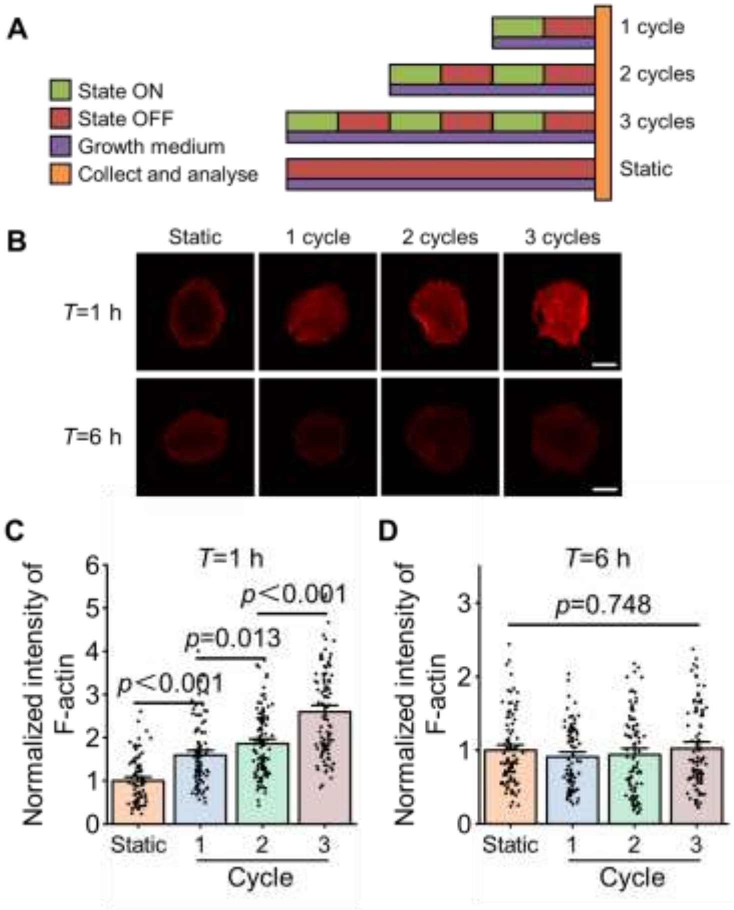

Figure S21. Residual F-actin within cells in the "OFF" state versus number and period of cycles. A. hMSCs were cultured on the hydrogels in growth media (purple) to experience various number (1, 2 or 3) and period (1 h or 6 h) of cycles of the "ON" (green)/ "OFF" (red) transitions. Control samples were cultured on the continuous "OFF" state in growth media without experiencing cyclic adhesion (static). **B**. The F-actin immunostaining images in hMSCs with *T* for 1 h and 6 h. **C**. Relative intensity was quantified based on the F-actin staining with *T* for 1 h (from left to right n = 84, 92, 104, 97 cells, p values were obtained using one-way ANOVA followed by Tukey's post hoc test, error bars are mean ± s.e.m). **D**. Relative intensity was quantified based on the F-actin staining with *T* for 6 h (from left to right n= 95, 87, 91, 88 cells, p values were obtained using one-way ANOVA followed by Tukey's post hoc test, error bars are mean ± s.e.m). Scale bars, 10 µm.

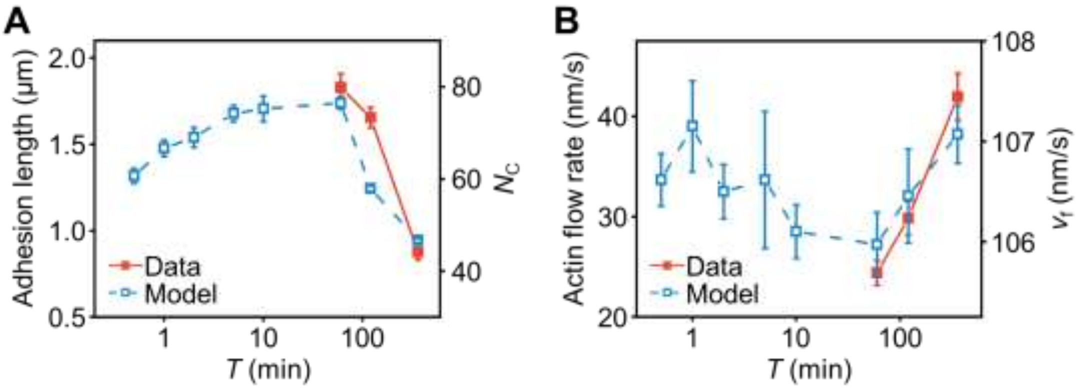

Figure S22. Cyclic integrin-mediated adhesion regulates the size of focal adhesions and actin flow rate. Comparison of model predictions (blue dashed line) and experimental characterization (red solid line) of adhesion length (**A**) and actin flow rate (**B**) as a function of cyclic period (*T*).

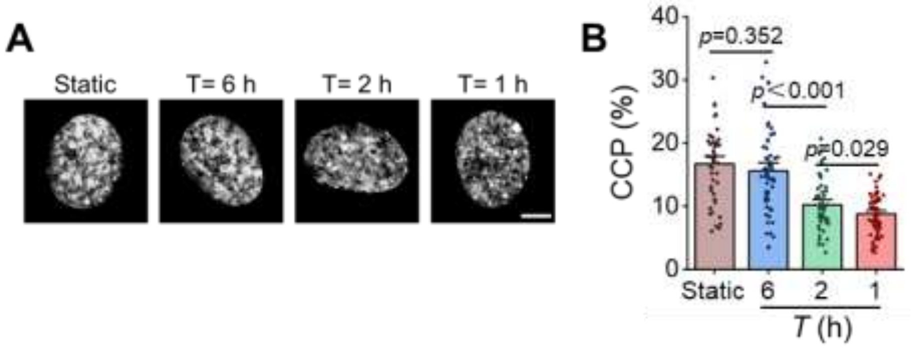

Figure S23. Cyclic integrin-mediated adhesion regulates chromatin condensation. A. Chromatin condensation of cells after static or three cycles of adhesions for T = 6, 2, 1 h as visualized by DAPI staining. (Scale bar, 5 μm) **B**. Corresponding quantification of the chromatin condensation parameter (CCP) for the same conditions in (A) (from left to right: n = 48, 59, 54, 64 cells, p values were obtained using one-way ANOVA followed by Tukey's post hoc test, error bars are mean ± s.e.m).

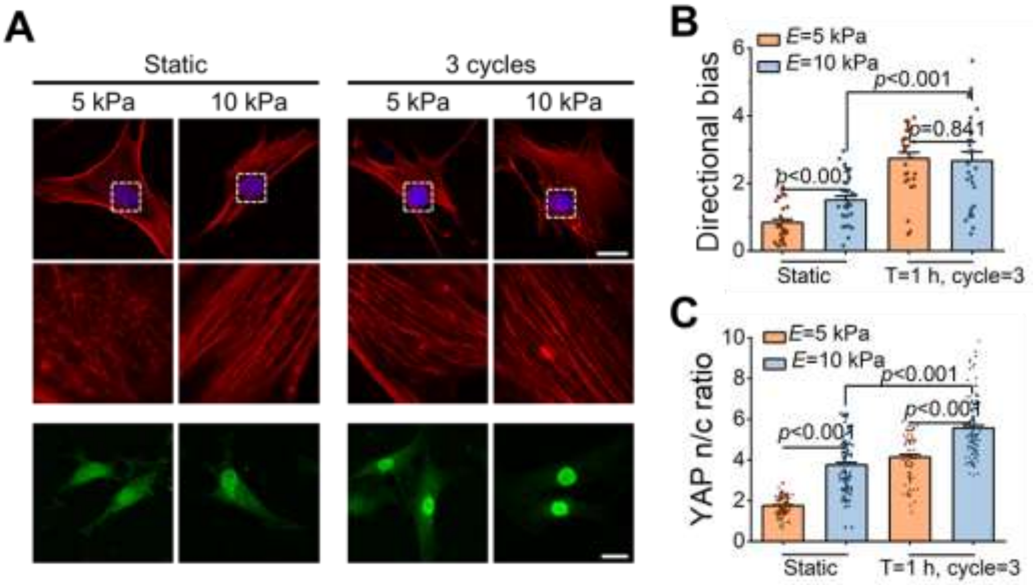

Figure S24. Increasing stiffness promotes F-actin alignment and YAP nuclear localization as predicted by the model. A. Representative images of F-actin (red) and YAP (green) in hMSCs for static or three cycles of adhesions (T = 1 h) on the 5 kPa and 10 kPa substrates. **B**. Corresponding quantifications of directional bias of actin (from left to right: n = 34, 29, 31, 28 cells, p values were obtained using one-way ANOVA followed by Tukey's post hoc test, error bars are mean ± s.e.m). **C**. Corresponding quantifications of YAP n/c ratios (from left to right: n = 112, 99, 75, 116 cells, p values were obtained using one-way ANOVA followed by Tukey's post hoc test, error bars are mean ± s.e.m). Scale bar, 20 μm.

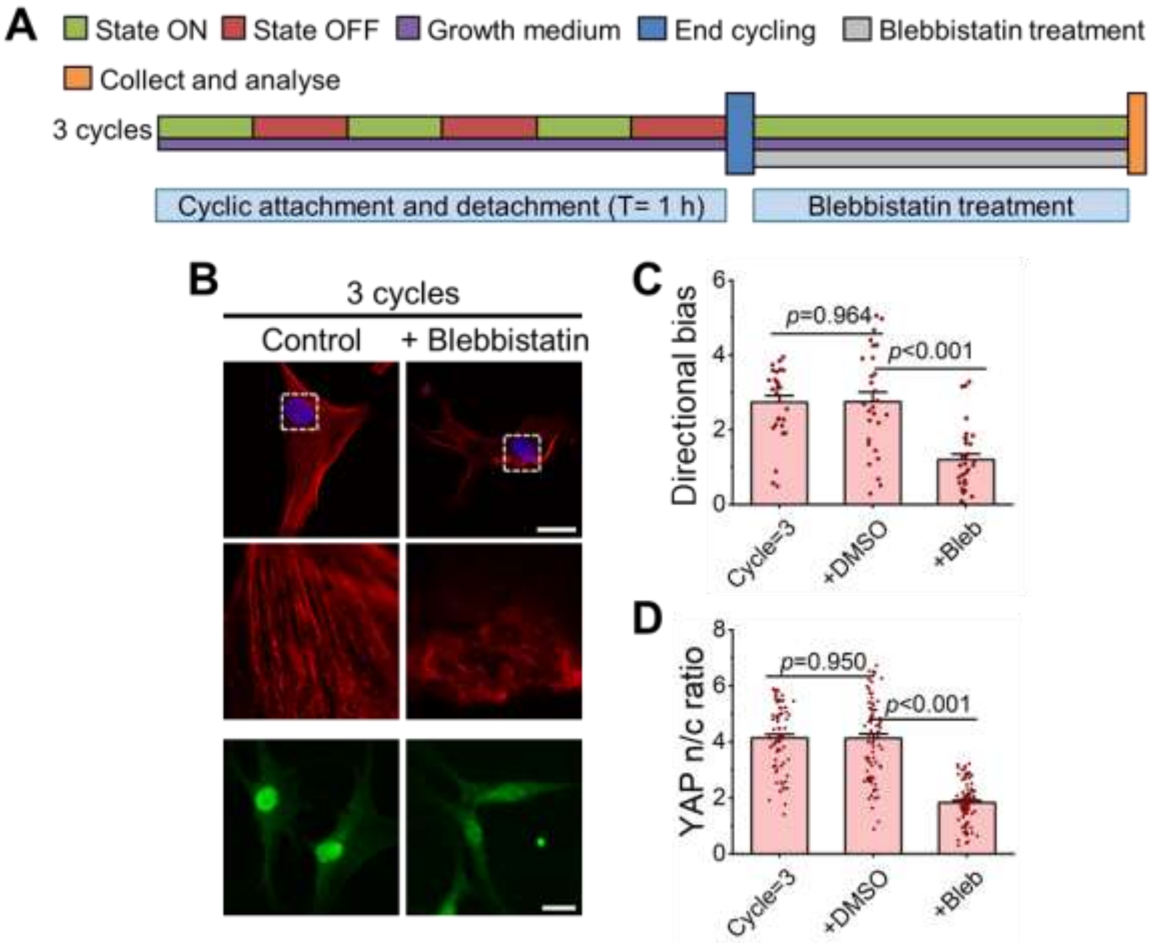

Figure S25. Decreasing myosin contractility inhibits F-actin alignment and YAP nuclear localization as predicted by the model. A. Schedules for three cycles of adhesions ($T = 1$ h) with or without blebbistatin treatment. **B**. Representative images of F-actin (red) and YAP (green) in hMSCs as the same schedules in (A). **C**. Corresponding quantifications of directional bias of F-actin (from left to right: n = 29, 28, 32 cells, p values were obtained using one-way ANOVA followed by Tukey's post hoc test, error bars are mean ± s.e.m). **D**. Corresponding quantifications of YAP n/c ratios (from left to right: n = 75, 78, 87 cells, p values were obtained using one-way ANOVA followed by Tukey's post hoc test, error bars are mean ± s.e.m). Scale bar, 20 µm.

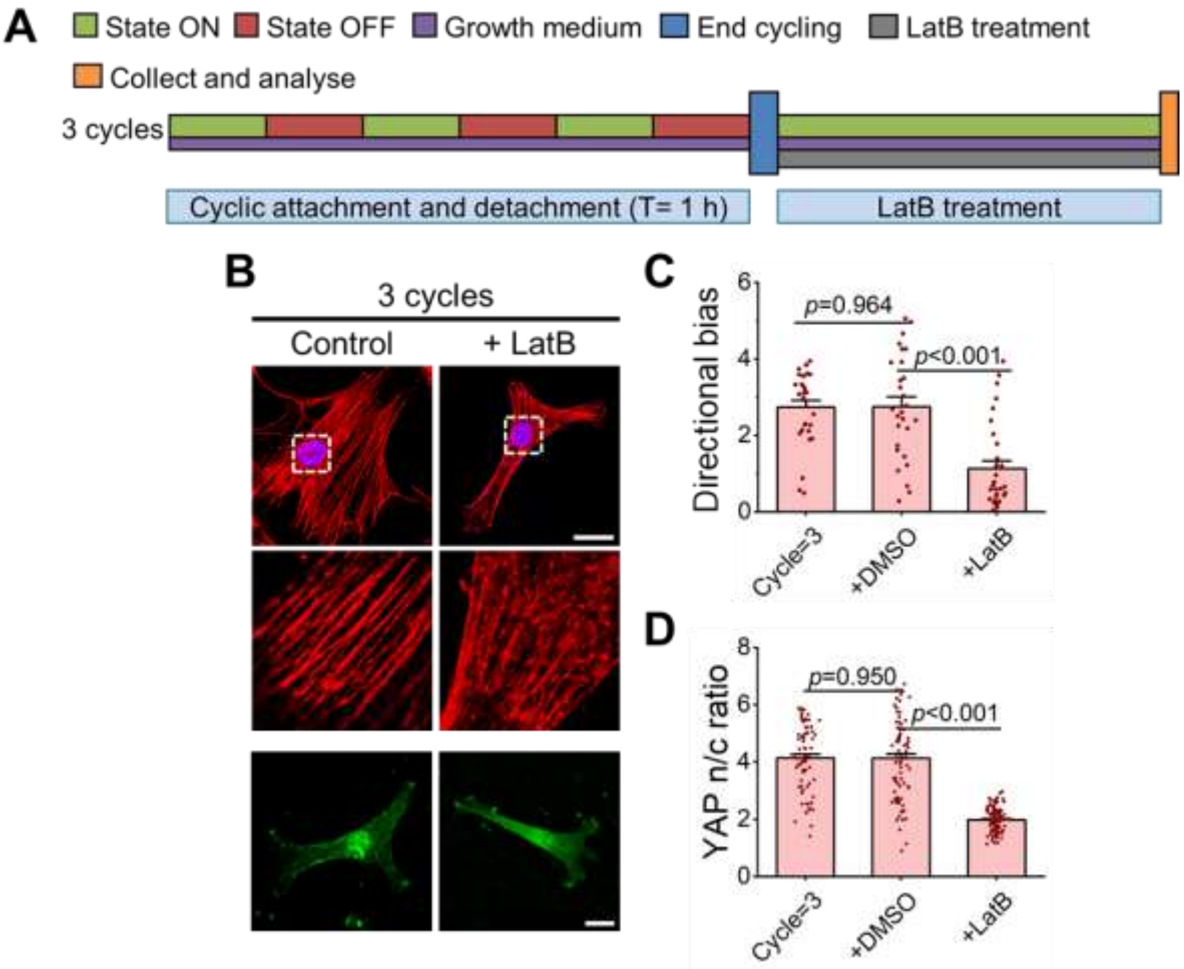

Figure S26. Increasing actin depolymerization rate inhibits F-actin alignment and YAP nuclear localization as predicted by the model. A. Schedules for three cycles of adhesions (T = 1 h) with or without LatB treatment. **B**. Representative images of F-actin (red) and YAP (green) in hMSCs as the same schedules in (A). **C**. Corresponding quantifications of directional bias of F-actin (from left to right: n = 29, 28, 31 cells, p values were obtained using one-way ANOVA followed by Tukey's post hoc test, error bars are mean ± s.e.m). **D**. Corresponding quantifications of YAP n/c ratios (from left to right: n = 75, 78, 115 cells, p values were obtained using one-way ANOVA followed by Tukey's post hoc test, error bars are mean ± s.e.m). Scale bar, 20 µm.

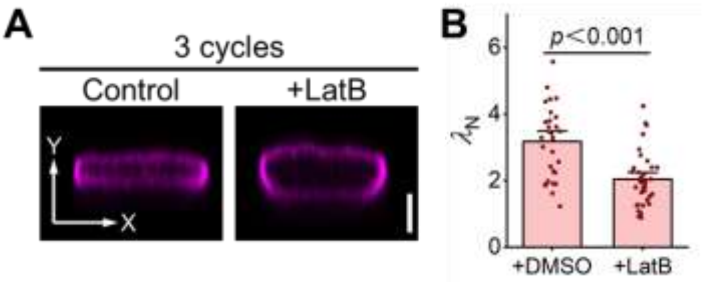

Figure S27. Inhibiting formation of F-actin reverses cyclic adhesion-enhancement of nuclear deformation. A-B. Lamin A/C staining (**A**) and quantification (**B**) of nuclear flattening for the same conditions in Figure S12A (from left to right: n = 29, 36 cells, p values were obtained using one-way ANOVA followed by Tukey's post hoc test, error bars are mean ± s.e.m). Scale bar, 5 µm.

Supporting Tables

Table S1: Sequences of all DNA strands used. Names and sequences of DNA strands used. Abbreviations: SH= thiol; N_3= azide.

Strand name	DNA sequence (5' to 3')
immobilizing strand	SH-ATATTGTTTGTTACACGGGATCCCGATTTT
bioactive strand	N_3-ATTTGAAACGAAAATCGGGATCCCGTGTAA
scrambled bioactive strand	N_3-TCAAGCTTAGATGTAACGAGACTAGGCATA
displacement strand	TTACACGGGATCCCGATTTTCGTTT

Table S2: List of input parameter values for CADS.

Symbol	Parameter Description	Value	Ref
N_{m}	Total number of motors	8,000	[2]
N_{c}	Total number of clutches	8,000	[2]
A_{tot}	Total possible actin protrusion length	10^6 nm	[2]
$k_{\mathrm{on}}^{\mathrm{act}}$	The rate constant of actin subunit adding to the F-actin module	20 nm/s	[4]
$k_{\mathrm{off}}^{\mathrm{act}}$	The rate constant that actin subunit disassociates from the F-actin module	40 nm/s	[4]
v_{dp}^{*}	The minimum rate of depolymerization	0-40 nm/s	Adjusted
p_0	The probability to find the FH2 dimer	0.56	[4]
a	The fraction of module traction force experienced by formin	0.06	Adjusted
δ	The size of actin monomer	5.4 nm	[4]
k_{mod}^{*}	Maximum module birth rate	0.1 s^{-1}	[2]
k_{cap}	Module capping rate	0.001 s^{-1}	[2]
l_{in}	Initial length of F-actin module	10,000 nm	Adjusted
l_{min}	Minimum length of F-actin module	1000 nm	Adjusted
l_{min}	Maximum length of F-actin module	40,000 nm	[2]
κ_{cell}	Cell spring constant	10^4 pN/nm	[2]
$n_{\mathrm{c,cell}}$	Number of cell body clutches	100	[2]
n_{m}^{*}	Maximum number of module motors	N_{m}*0.01	Adjusted
F_{m}	Motor stall force	2 pN	[2]
$\mathrm{v}_{\mathrm{f}}^{*}$	Unloaded motor velocity	110 nm/s	[2]
n_{c}^{*}	Maximum number of module clutches	N_{c}*0.01	Adjusted
k_{on}	Clutch on-rate	0.11 s^{-1}	[13]
k_{off}^{*}	Clutch unloaded off-rate	0.1 s^{-1}	[2]
F_{b}	Characteristic clutch rupture force	6 pN	[2]
k_{a}	Rate constant for myosin filament formation	0.014 s^{-1}	Adjusted
k_{d}	Rate constant for myosin filament disassembly	0.0014 s^{-1}	Adjusted

Supporting Reference

[1] C. E. Chan, D. J. Odde, *Science* **2008**, 322, 1687.

[2] B. L. Bangasser, G. A. Shamsan, C. E. Chan, K. N. Opoku, E. Tüzel, B. W. Schlichtmann, J. A. Kasim, B. J. Fuller, B. R. McCullough, S. S. Rosenfeld, D. J. Odde, *Nature Communications* **2017**, 8, 15313.

[3] a) E. Kassianidou, D. Probst, J. Jager, S. Lee, A.-L. Roguet, U. S. Schwarz, S. Kumar, *Cell Reports* **2019**, 27, 1897; b) A. K. Paknikar, B. Eltzner, S. Köster, *Progress in Biophysics and Molecular Biology* **2019**, 144, 166; c) C.-K. Huang, A. Donald, *Journal of the Royal Society Interface* **2015**, 12, 20141064.

[4] A. Jegou, M. F. Carlier, G. Romet-Lemonne, *Nature Communications* **2013**, 4, 1883.

[5] T. Vignaud, C. Copos, C. Leterrier, M. Toro-Nahuelpan, Q. Tseng, J. Mahamid, L. Blanchoin, A. Mogilner, M. Théry, L. Kurzawa, *Nature Materials* **2020**, 3, 410.

[6] a) E. McEvoy, V. S. Deshpande, P. McGarry, *Biomechanics and Modeling in Mechanobiology* **2019**, 18, 921; b) V. S. Deshpande, R. M. McMeeking, A. G. Evans, *Proceedings of the National Academy of Sciences* **2006**, 103, 14015; c) W. Ronan, V. S. Deshpande, R. M. McMeeking, J. P. McGarry, *Biomechanics and Modeling in Mechanobiology* **2014**, 13, 417.

[7] A. Elosegui-Artola, R. Oria, Y. Chen, A. Kosmalska, C. Pérez-González, N. Castro, C. Zhu, X. Trepat, P. Roca-Cusachs, *Nature Cell Biology* **2016**, 18, 540.

[8] a) Z. Wei, V. S. Deshpande, R. M. McMeeking, A. G. Evans, *Journal of Biomechanical Engineering* **2008**, 130, 031009; b) E. L. Elson, G. M. Genin, *Experimental Cell Research* **2013**, 319, 2490.

[9] B. Cheng, M. Lin, Y. Li, G. Huang, H. Yang, G. M. Genin, V. S. Deshpande, T. J. Lu, F. Xu, *Biophysical Journal* **2016**, 111, 2051.

[10] a) M. Vicente-Manzanares, X. Ma, R. S. Adelstein, A. R. Horwitz, *Nature Reviews Molecular Cell Biology* **2009**, 10, 778; b) M. Garrido-Casado, G. Asensio-Juárez, M. Vicente-Manzanares, *Annual Review of Cell and Developmental Biology* **2021**, 37, 285.

[11] L. S. Tobacman, E. D. Korn, *Journal of Biological Chemistry* **1983**, 258, 3207.

[12] a) W. Luo, C.-h. Yu, Z. Z. Lieu, J. Allard, A. Mogilner, M. P. Sheetz, A. D. Bershadsky, *Journal of Cell Biology* **2013**, 202, 1057; b) J. Lohner, J.-F. Rupprecht, J. Hu, N. Mandriota, M. Saxena, D. P. de Araujo, J. Hone, O. Sahin, J. Prost, M. P. Sheetz, *Nature Physics* **2019**, 15, 689.

[13] C. Zhang, H. Y. Zhu, X. R. Ren, B. Gao, B. Cheng, S. B. Liu, B. Y. Sha, Z. Q. Li, Z. Zhang, Y. Lv, H. H. Wang, H. Guo, T. J. Lu, F. Xu, G. M. Genin, M. Lin, *Nature Communications* **2021**, 12, 6229.
